# Ten new genera of Agathidini (Hymenoptera, Braconidae, Agathidinae) from Southeast Asia

**DOI:** 10.3897/zookeys.660.12390

**Published:** 2017-03-13

**Authors:** Michael J. Sharkey, Eric Chapman

**Affiliations:** 1 Department of Entomology, University of Kentucky, S225 Agricultural Science Center North, Lexington, KY, 40546-0091, USA

**Keywords:** Taxonomy, systematics, *Agathigma*, *Asperagathis*, *Chimaeragathis*, *Cymagathis*, *Liragathis*, *Leuroagathis*, *Scabagathis*, *Trochantagathis*, *Xanthagathis*, *Zosteragathis*

## Abstract

The Agathidini (Braconidae: Agathidinae) genera of Southeast Asia are revised based on a phylogenetic analysis of COI and 28S. Ten new genera are proposed, i.e., *Agathigma*, *Asperagathis*, *Chimaeragathis*, *Cymagathis*, *Liragathis*, *Leuroagathis*, *Scabagathis*, *Trochantagathis*, *Xanthagathis*, and *Zosteragathis*. An illustrated key to the Southeast Asian genera of this tribe is presented. Species from Thailand are keyed and described for all genera of Agathidini except *Bassus* and *Zosteragathis* which have too many species for this publication and will be dealt with separately. The phylogenetic analyses indicate that *Bassus* *s.s.* is polyphyletic. However, there are no morphological characters to support this and we have retained the current concept of *Bassus*, which is basically those Agathidini with simple tarsal claws. Numerous new combinations are proposed based on species that are moved to the newly erected genera.

## Introduction

Agathidinae is a moderately diverse subfamily of Braconidae with about 1,200 described species (Yu et al. 2012) and many times that number are yet to be named. Larvae are parasitoids of lepidopteran caterpillars of a multitude of families. Most agathidine genera, and probably all of the genera treated here, attack an early instar caterpillar and are quiescent until the host has reached the final instar and is ready to spin a cocoon. At this point in time the parasitoid larva becomes active and quickly consumes the host, i.e., they are koinobiont endoparasitoids.

This is the sixth publication on the Agathidine fauna with a concentration on Thailand. Sharkey et al. (2009) revised the Oriental genera of Agathidinae. Sharkey and Clutts (2011) revised the Thai agathidine genera with one or a few species and updated the generic key to the Oriental genera. Sharkey and Stoelb (2012, 2013) revised the Thai species of *Zelodia* van Achterberg and *Agathacrista* Sharkey. Lastly, van Achterberg et al. (2014) revised the Thai species of *Euagathis*. It is the aim of this paper to revise the genera of Agathidini that have not been treated and to describe the Thai species of these that are not overly species-rich.

The recent redefinition of *Bassus* (Sharkey et al. 2009) to refer only to those species of Old World agathidines with simple claws, necessitated the erection or resurrection of numerous genera to house species formerly contained in the broader, polyphyletic concept of *Bassus* (Sharkey et al. 2015; Sharkey and Chapman 2015; Sharkey and Stoelb 2013; Sharkey et al. 2016; Achterberg and Long 2010). The previously published genera of this nature are: *Gelastagathis* Sharkey, 2015; *Aphelagathis* Sharkey, 2015; *Pneumagathis* Sharkey, 2015; *Agathacrista* Sharkey, 2013; *Neothlipsis* Sharkey, 2011; *Gyragathis* Achterberg & Long, 2010; *Aerophilus* Szépligeti,1902; and *Therophilus* Wesmael, 1837. Most of the aforementioned genera, including *Bassus* *s.s.*, are small and restricted to the Old or New world. The two exceptions are *Aerophilus*, and *Therophilus*. These are both species-rich and cosmopolitan. Unfortunately, but perhaps necessarily, *Therophilus* has become the new dumping ground for unplaced members of Agathidini (Stevens et al. 2010, 2011; Achterberg and Long, 2010). This is all the worse because most of the species recently placed in *Therophilus* are not closely related to it. *Therophilus* is sister to the clade *Mesocoelus* + *Aneurobracon* and has a number of unique features as outlined in Sharkey and Stoelb (2012). It is the purpose of this paper to erect new Old World genera to avoid the further debasement of *Therophilus*. The revision is primarily based on material collected in Thailand. New species from Thailand are keyed and described for all genera of Agathidini except *Bassus* and *Zosteragathis* which have too many species for this publication and will be dealt with separately.

## Methods

All specimens except for some duplicates are deposited in the Entomological Museum of The Queen Sirikit Botanic Gardens, Chaing Mai, Thailand.

### Diagnoses

Diagnoses are rather comprehensive however an abbreviated diagnosis for each genus is given in bold font within each diagnosis.

### Morphological terms

Morphological terms are from Sharkey and Wharton (1997) and are matched to the Hymenoptera Anatomy Ontology (HAO; Yoder et al. 2010; http://portal.hymao.org/projects/32/public/ontology/). Identifiers (URIs) in the format http://purl.obolibrary.org/obo/HAO_XXXXXXX represent anatomical concepts in HAO version http:// purl.obolibrary.org/obo/hao/2011-05-18/hao.owl. They are provided to enable readers to confirm their understanding of the anatomical structures being referenced. To find out more about a given structure, including images, references and other metadata, use the identifier as a web-link, or use the HAO:XXXXXXX (note colon replaces underscore) as a search term at http://glossary.hymao.org. In this paper, terms are linked to the ontology in the results section, each couplet of the key, and in the first description of a taxon (genus *Aphelagathis*). From this point forward, only terms that do not appear in these areas are hyperlinked.

### DNA extraction, PCR and sequencing

DNA was extracted from individual legs with the QIAGEN DNeasy Blood and Tissue Kit using the animal tissue protocol (QIAGEN Inc., Chatsworth, California, USA). The nuclear 28S, regions D2-D3 (~600 bp), rDNA and mitochondrial COI (~650 bp) genes were amplified with the 28S primer pairs 28SD2F (Belshaw and Quicke 1997) and D3R (Harry et al. 1996) and the COI primer pairs LepF1 and LepR1 (Hebert et al. 2004). For COI, PCR was conducted using Takara reagents, with each reaction consisting of 1X buffer, 0.3 mM nucleotides, 0.4 μM of each primer, 0.625 U Takara Ex Taq, ddH2O, and 1–3 μL template DNA in a total reaction volume of 25 μL. The thermal cycling protocol had an initial denaturation period at 95 °C for 2.5 min, followed by 40 cycling steps which denatured at 95 °C for 30 s, annealed at 44 °C for 30 s and extended at 68 °C for 45 s, with a final extension step of 72 °C for 7 min. For 28S, PCR consisted of Qiagen 1X buffer, 4 mM MgSO4, 0.3 mM dNTP, 0.4 μM of each primer, 1.0 U Qiagen Taq, ddH2O, and 1–3 μL template DNA with a total reaction volume of 25 μL. Thermal cycling was as above except annealing at 53 °C, extending for 70 s, and a total of 35 cycles. To determine reaction success, PCR products were electrophoresed in 1% agarose stained with ethidium bromide. PCR products were outsourced for Sanger sequencing either by the Advanced Genetic Technologies Center (University of Kentucky, Lexington, KY) or Beckman Coulter Genomics (Danvers, MA, USA) using labelled dideoxy-nucleotides with ABI 3730, Big-Dye Terminator mix v. 3.0 or with ABI PRISM 3730xl, BigDye Terminator mix v. 3.1 (Applied Biosystems, Foster City, California, USA).

### DNA assembly and phylogenetic analysis

Bi-directional sequences were aligned and edited using Geneious Pro (v. 6.1.5; Drummond et al. 2009) and multiple alignments were assembled using MAFFT (v. 5; Katoh et al. 2006) using the default settings and refined by eye. Maximum likelihood (ML) phylogenetic analyses were conducted on a concatenated (using MacClade v. 4.08; Maddison and Maddison 2000) 1,313-character total evidence data set (COI = 723 bp, 28S = 590 bp) using Garli (v. 2.01; Zwickl 2006). The data were partitioned by gene region and codon position (COI: 3 partitions; 28S: unpartitioned, total of 4 partitions). We applied the most complex model available (GTR+I+G; Rodriguez et al. 1990) to each partition as per recommendations of Huelsenbeck and Rannala (2004). We conducted a 20-replicate ML search for the tree of highest log-likelihood and a 500-replicate ML bootstrap analysis (Felsenstein 1985). Both analyses used the default settings. The data sets analyzed herein are available from the authors upon request.

## Results

### Phylogenetic considerations

Here we treat a number of species from Thailand and propose 10 new genera. Most of these are demonstratively monophyletic and morphologically distinct; however, some compromises are made due to poor resolution in the phylogenetic analysis. The tree of highest log-likelihood is presented in Figure 1, with the ML bootstrap values plotted on nodes with ≥50% bootstrap support.

**Figure 1.**
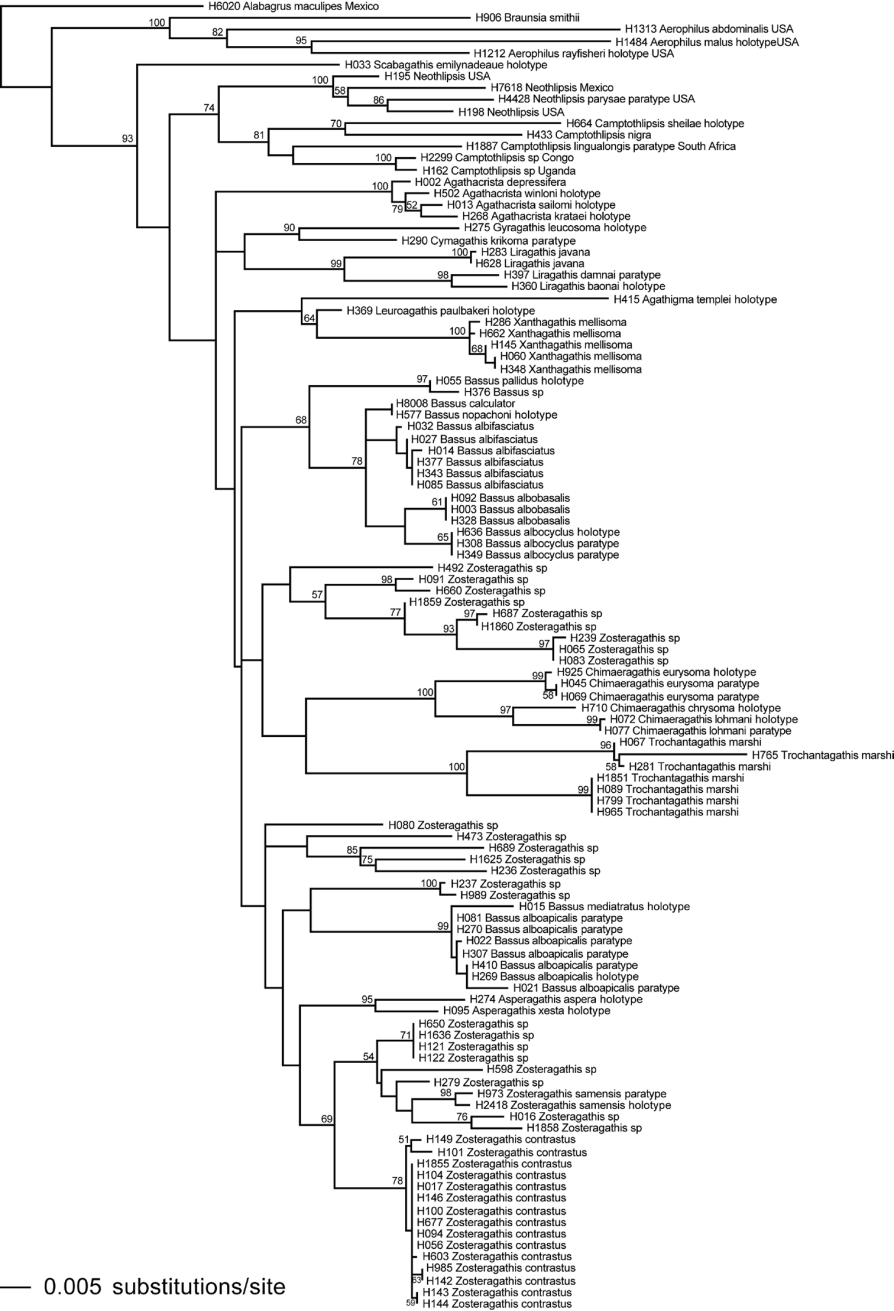
Tree of highest log-likelihood from 20 ML search reps of the combined COI+28S data set with bootstrap values ≥50% plotted at the nodes.

In the case of the Thai fauna treated here, there were a number of options in terms of the number of genera that could be proposed. The criteria that I (MJS) used in making decisions on generic limits were: to recognize those monophyletic clades with high (usually > 90%) ML bootstrap support values (Fig. 1), which are also clearly diagnosed by morphological character states, and the recognition of which would not render other genera paraphyletic. A secondary criterion was to rely solely on potential morphological synapomorphies when they were not contradicted by molecular evidence, as in the case of *Bassus* (see below). Most of the genera are well-supported by molecular evidence as demonstrated in Figure 1 and by morphological synapomorphies; e.g., *Agathacrista, Asperagathis*, *Camptothlipsis*, *Chimaeragathis*, *Liragathis*, *Neothlipsis*, and *Trochantagathis*. However, *Bassus* was polyphyletic forming two clades. The members of clades are not distinct morphologically, and they share the character state of lacking a lobe at the base of the tarsal claws. Rather than dividing *Bassus* into two indistinguishable genera, we prefer to continue to recognize the current concept. *Agathigma*, *Cymagathis*, *Gyragathis*, *Leuroagathis*, *Scabagathis*, and *Xanthagathis* are each represented by only one species; therefore there are no nodes from which to obtain bootstrap values. However, all are on relatively long branches on the total evidence tree, all have distinct autapomorphies, and none renders another taxon paraphyletic (Fig. 1). *Zosteragathis* is most likely a paraphyletic genus. Although members have similarities, there is not a morphological or molecular autapomorphy for the group. The ML bootstrap values (plotted on Fig. 1) support multiple monophyletic clades of *Zosteragathis*, but none of these have an obvious morphological autapomorphy. Rather than propose a number of vague genera, I (MJS) thought it best to propose a conservative hypothesis in the interest of stability.

### Discussions of each genus are presented below in alphabetical order

***Agathacrista* Sharkey, 2013**: The genus was described and revised by Sharkey and Stoelb (2013). The thin interantennal crest is an autapomorphy for the genus, though convergently found in *Chimaeragathis* and in a few species of a few other genera; e.g., *Therophilus*.

***Agathigma* Sharkey, new genus**: *Agathigma templei* is the sole species. Morphological autapomorphies are the squared temples (Fig. 2) and the labial palpus reduced to 2 segments. The former character state is rarely found in other agathidine genera such as *Gyragathis* and convergently in a few New World *Therophilus* and *Aerophilus*. In the tree in Figure 1 it appears as sister to the clade *Leuroagathis* + *Xanthagathis*; however there is no bootstrap support for this relationship. The branch leading to the terminus *Agathigma templei* is the longest of all branches, a fact that further erodes confidence in its placement.

***Asperagathis* Sharkey, new genus**: This genus is sister to one of the *Zosteragathis* clades in the total evidence tree (Fig. 1). The rugose sculpture of the mesosoma is a proposed autapomorphy; however, even rougher sculpture occurs in Southeast Asian specimens that author MJS has viewed which may not be congeneric.

***Bassus* Fabricius, 1804**: *Bassus*, the strict definition of which was proposed by Sharkey et al. (2009), is restricted to those Old World agathidines with simple tarsal claws. This autapomorphy is convergently found in all *Sesioctonus*, a Neotropical genus, and in a few species of other genera such as *Neothlipsis*. *Bassus* is polyphyletic in the ML tree (Fig. 1); and the polyphyly is not resolved in the ML bootstrap tree (not shown). Interestingly, both COI and 28S gene-trees (analyzed as above; not shown) are completely congruent with the tree in Figure 1 regarding *Bassus*, showing the same species membership in the 2 clades. Because there are no obvious morphological character states to distinguish either of the two clades of *Bassus*, we choose to retain the genus as it is until more data confirm that it is not monophyletic.

***Camptothlipsis* Enderlein, 1920**: This is an Old World genus, primarily tropical, that is sister to the New World genus *Neothlipsis* in Figure 1 (but see Fig. 1 in Sharkey and Chapman 2015). Both lack strong sculpture on the metasomal median tergites and possess granulate sculpture on metasomal median tergites 1–3.

***Chimaeragathis* Sharkey, new genus**: An interantennal crest is shared convergently with members of *Agathacrista*. Another autapomorphy is the relatively dense pilosity on the scutellar triangle and the lateral areas of the propodeum. The total evidence tree (Fig. 1) shows a sister-group relationship with ((*Gyragathis* + *Cymagathis*) *Liragathis*) but this relationship lacks bootstrap support.

***Cymagathis* Sharkey, new genus**: An autapomorphy for the genus is that the second median tergite is covered with strong smooth striae that end evenly at the apex of the tergite with the striae forming a semicircular pattern anteromedially. This is convergently found in some species of *Trochantagathis*. It is sister to *Gyragathis* on the total evidence tree (Fig. 1), supported with a bootstrap value of 90.

***Gyragathis* Achterberg & Long, 2010**: An autapomorphy for the genus is that the antennal sockets are margined with carinae. Other possible autapomorphies include the interantennal space with a longitudinal depression and the squared temples, the latter of which is shared convergently with *Agathigma*. Possession of margined antennal sockets is a character state shared by several distantly related New World Agathidini genera, e.g., *Alabagrus* and *Trachagathis*, as well as some genera of Cremnoptini and Disophrini. *Gyragathis* is sister to *Cymagathis* on the total evidence tree (Fig. 1), supported with a bootstrap value of 90.

***Leuroagathis* Sharkey, new genus**: This genus possesses two autapomorphic character states: notauli absent, and median tergite 1 smooth, lacking sculpture. It is sister to *Xanthagathis* in the total evidence tree but the relationship lacks bootstrap support (Fig. 1). Many agathidines from Australia share these two autapomorphies. The one Australian specimen with these characteristics for which we obtained 28S and COI data does not fall within the clade examined herein (unpublished).

***Liragathis* Sharkey, new genus**: An autapomorphy is the median carina of the first median tergite which is as strong as, or stronger than, the lateral carinae. It is sister to *Gyragathis* + *Cymagathis* but this relationship lacks bootstrap support (Fig. 1).

***Scabagathis* Sharkey, new genus**: There are two autapomorphic character states. The vertex has rough sculpture and the labial palpus, normally 4-segmented, is 3-segmented, with the third palpomere lacking. The total evidence tree (Fig. 1) shows this genus arising early in the evolution of this group and is sister to a clade containing all genera except *Aerophilus*, *Alabagrus*, and *Braunsia*.

***Xanthagathis* Sharkey, new genus**: It is sister to *Leuroagathis* in the total evidence tree, but the relationship has a low bootstrap support (Fig. 1; bootstrap value = 64). The pale coloration (particularly the yellow head) is autapomorphic. Other potential autapomorphic states are the hyaline wings and the smooth second median tergite.

***Zosteragathis* Sharkey, new genus**: There are no obvious morphological synapomorphies for *Zosteragathis* and its monophyly is not supported (Fig. 1). Most species have fine longitudinal striations on the second metasomal median tergite and most have a white transverse band on the same tergite. Neither of these is universal and the striations are found in other genera. Members of *Zosteragathis* are recovered in five separate clades in the total evidence tree (Fig. 1). Monophyly of the genus is not falsified in the total evidence bootstrap tree (not shown) where seven *Zosteragathis* clades fall into a large polytomy that includes all genera in the tree except *Aerophilus*, *Alabagrus*, and *Braunsia*. Although monophyly of *Zosteragathis* is dubious, it seems preferable to the alternative of erecting new genera for weakly supported clades that have little or no morphological or sequence support.

### Key to Thai genera of Agathidini

**Table:** 

1	**A1.** Fore and mid claws cleft. **A2.** Ovipositor variable, often barely exerted or shorter than 1/2 length of metasoma, rarely longer.	**Disophrini** and **Cremnoptini**
–	**B1.** Fore and mid claws simple with or without a basal lobe. **B2.** Ovipositor longer than 1/2 length of metasoma	**2**
2	**A.** Median tergite 1 entirely smooth	**20**
–	**B.** Median tergite 1 mostly granulate or coriarious	**21**
–	**C.** Median tergite 1 with other sculpture, usually striate	**3**
3(2)	**A1.** Median tergite 3 usually extensively striate in anterior half or more. **A2.** Straight carina situated above hind coxal cavities (CC)	4
–	**B1.** Median tergite 3 not extensively striate, usually smooth, or rarely, weakly coriarious. **B2.** If carina exists between hind coxal cavities (CC) then it is curved and dipping below dorsal margin of coxal cavities.	**5**
4(3)	**A.** Adventitious vein (2RS) on r-m crossvein of fore wing absent or indicated only by slight swelling	***Aerophilus* Szépligeti**
–	**B.** Adventitious vein (2RS) on r-m crossvein of fore wing present & distinct	***Braunsia* Kriechbaumer**
5(3)	**A.** Fore and mid tarsal claws with a basal lobe	**6**
–	**B.** Fore and mid tarsal claws simple	***Bassus* Fabricius**
6(5)	**A.** Mouthparts long, galea significantly longer than wide; gena often elongate	***Agathis* Latreille**
–	**B.** Mouthparts short (normal), galea not longer than wide; gena not especially elongate	**7**
7(6)	**A.** With carina partly or completely surrounding antennal socket	***Gyragathis* Achterberg & Long**
–	**B.** Lacking carina partly or completely surrounding antennal socket	**8**
8(7)	**A.** Hind trochantellus with ventral longitudinal carinae	***Trochantagathis* gen. n.**
–	**B.** Hind trochantellus lacking ventral longitudinal carinae	**9**
9(8)	**A.** Vertex of head smooth, with weak punctures	**10**
–	**B.** Vertex of head rugosopunctate	***Scabagathis* gen. n.**
10(9)	**A1.** RS+M vein of fore wing mostly or entirely absent. **A2.** Notauli present	**11**
–	**B1.** RS+M vein of fore wing present and complete. **B2.** Notauli absent	***Earinus* Wesmael**
11(10)	**A.** Sharply declivous crest in interantennal space present	**12**
–	**B.** Sharply declivous crest in interantennal space absent	**14**
12(11)	**A.** Cub vein of hind wing absent, or if present, clear and weak, not tubular, and not contiguous with cu-a (base)	**13**
–	**B.** Cub vein of hind wing present, tubular and pigmented	***Therophilus anuchati* Sharkey**
13(12)	**A.** Scutellar triangle smooth with punctures and sparse setae	***Agathacrista* Sharkey**
–	**B.** Scutellar triangle rugose or with dense aciculations, sometimes obscured with dense setae	***Chimaeragathis* gen. n.**
14(11)	**A.** Median tergite 2 mostly striate with striae coming to an abrupt and uniform end at or near apex of tergite	**15**
–	**B.** Median tergite 2 striate or not; if striate, striae not coming to an abrupt and uniform end at apex of tergite	**17**
15(14)	**A.** Semicircular striae at base of median tergite 2 present	***Cymagathis* gen. n.**
–	**B.** Semicircular striae at base of median tergite 2 not present	**16**
16(15)	**A.** Pegs of fore tibia present (concolorous with tibia therefore difficult to see)	***Asperagathis* gen. n.**
–	**B.** Pegs of fore tibia absent	***Zosteragathis* gen. n.**
17(14)	With two or more of the following characters: **A1.** Rs vein of fore wing weak medially and bent: **A2.** Sclerite separating hind coxal cavities from metasomal foramen narrow or absent. **A3**. Apex of scutellum (at border with metanotum) with a distinct, often semicircular, depression. **A4**. Interantennal space often with two small protrusions separated by a depression (use frontal view). **A5**. Cub vein of hind wing pigmented and tubular where it is attached to subbasal cell and causing an angle in the distal margin of the cell where it is attached Note: often (70%) small and pale in coloration	***Therophilus* Wesmael**
–	With none or at most one of the above character states. Rather the following character states apply: **B1.** Rs vein of fore wing evenly sclerotized and straight. **B2.** Sclerite separating hind coxal cavities from metasomal foramen relatively wide. **B3**. Apex of scutellum (at border with metanotum) smooth or sculptured but lacking deep depression(s). **B4**. Interantennal space without two small protrusions separated by a depression (use frontal view), rather smooth, or with a median keel that may or may not be pronounced. **B5**. Cub vein of hind wing absent, OR not attached to basal cell, OR not pigmented, and subbasal cell not angled at point of intersection. Note: often (70%) small and pale in coloration Note: body usually mostly melanic	**18**
18(17)	**A.** Median tergite 2 partly or entirely white, ivory, or pale yellow	***Xanthagathis* gen. n.**
–	**B.** Median tergite 2 entirely melanic	**19**
19(18)	**A.** Temples rounded. **AA.** Median longitudinal ridge of first median tergite present	***Liragathis* gen. n.**
–	**B.** Temples squared. **BB.** Median longitudinal ridge of first median tergite absent	***Agathigma* gen. n.**
20(2)	**A.** RS+M vein of fore wing mostly or entirely absent	***Leuroagathis* gen. n.**
–	**B.** RS+M vein of fore wing present and complete	***Earinus* Wesmael**
21(2)	**A.** RS vein of fore wing completely absent	***Aneurobracon* Brues**
–	**B.** RS vein of fore wing present, though sometimes interrupted at midlength	***Camptothlipsis* Enderlein**

**Agathidini key:**
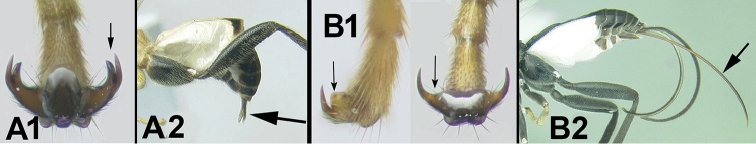
Figure 1

**Agathidini key:**
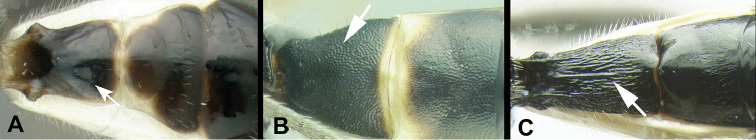
Figure 2

**Agathidini key:**
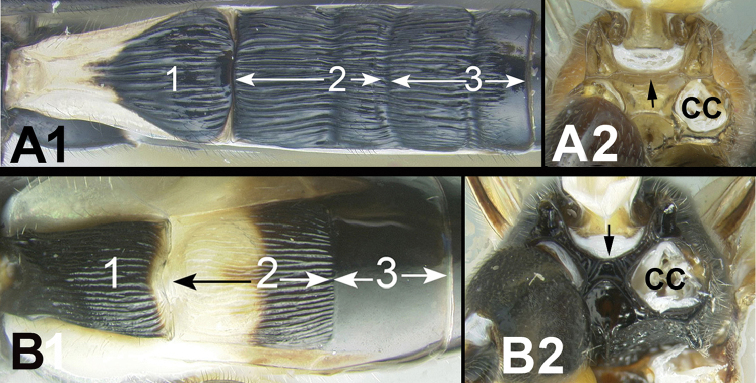
Figure 3

**Agathidini key:**
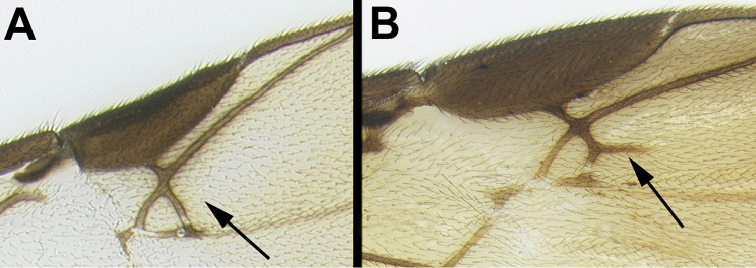
Figure 4

**Agathidini key:**
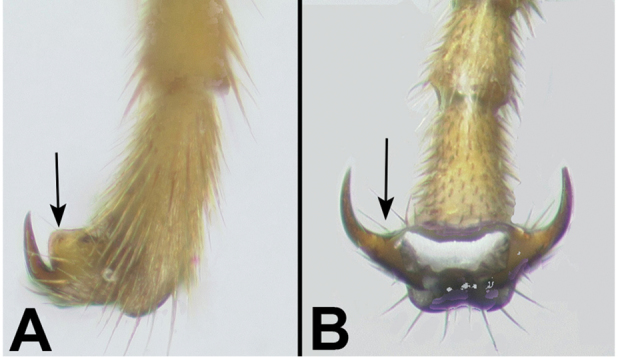
Figure 5

**Agathidini key:**
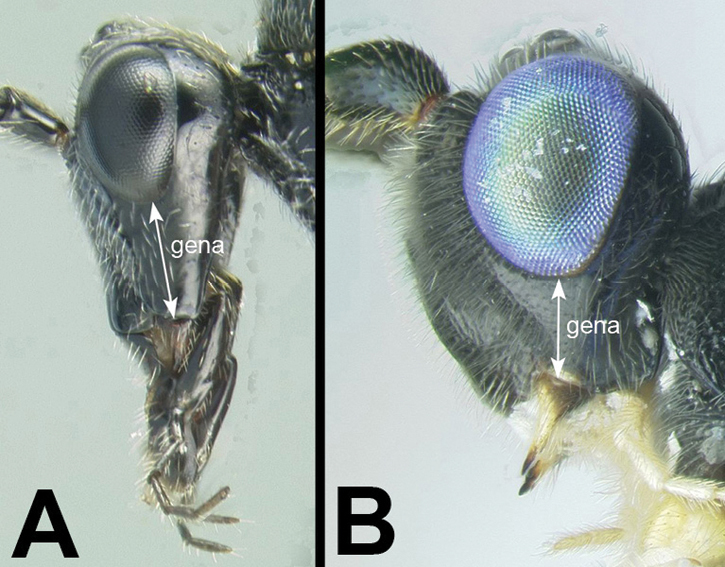
Figure 6

**Agathidini key:**
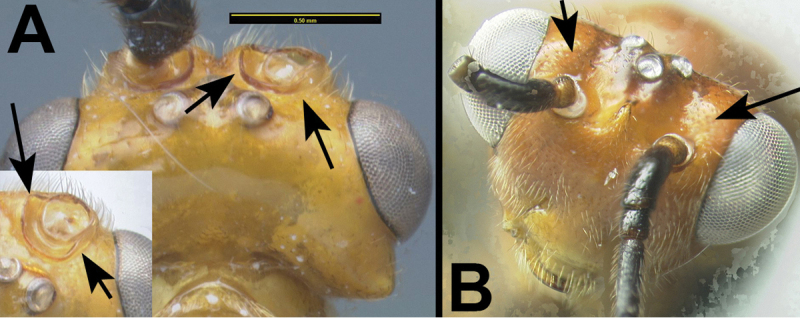
Figure 7

**Agathidini key:**
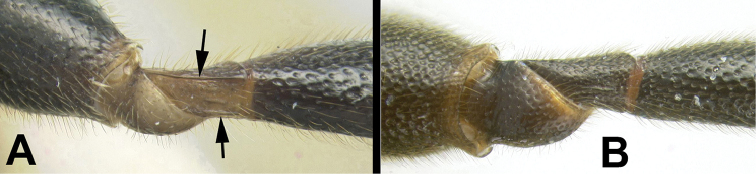
Figure 8

**Agathidini key:**
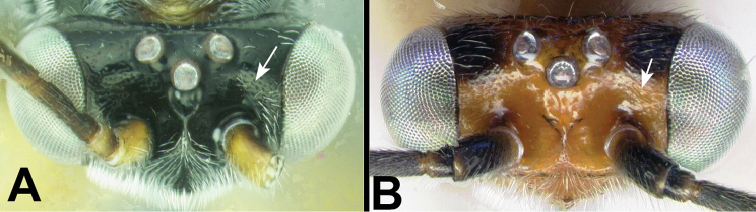
Figure 9

**Agathidini key:**
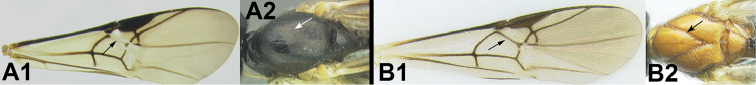
Figure 10

**Agathidini key:**
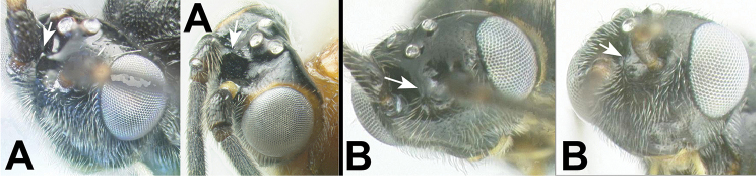
Figure 11

**Agathidini key:**
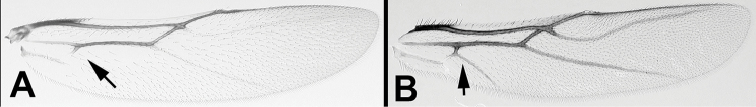
Figure 12

**Agathidini key:**
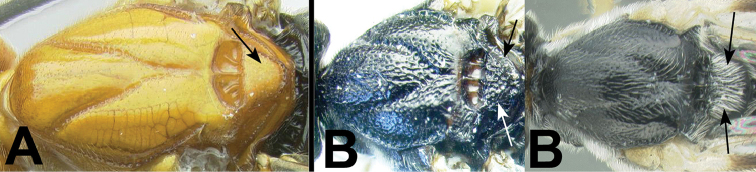
Figure 13

**Agathidini key:**
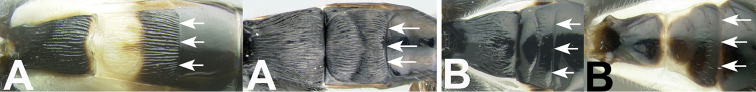
Figure 14

**Agathidini key:**
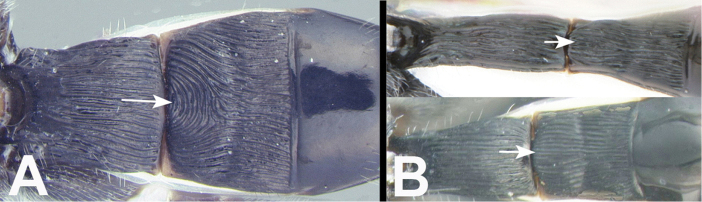
Figure 15

**Agathidini key:**
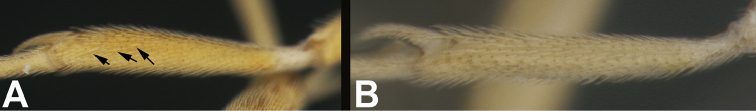
Figure 16

**Agathidini key:**
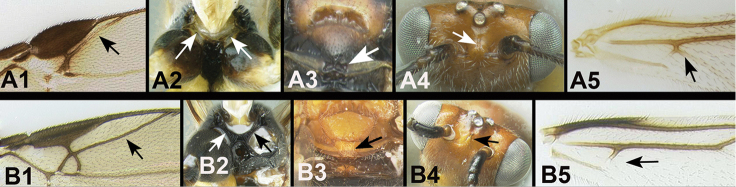
Figure 17

**Agathidini key:**
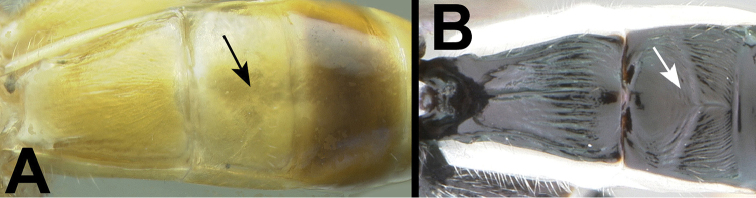
Figure 18

**Agathidini key:**
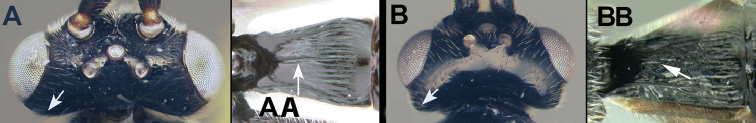
Figure 19

**Agathidini key:**
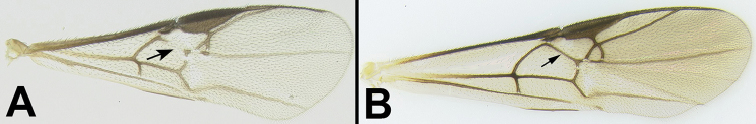
Figure 20

**Agathidini key:**
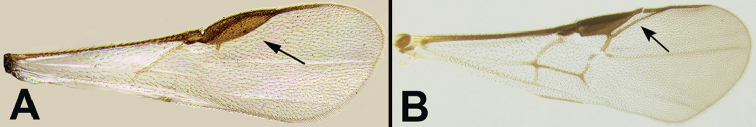
Figure 21

### Descriptions

Note: The text in bold font in the diagnoses below show a minimum set of character states to distinguish the taxon. The numbers preceded with the letter H are unique identifiers associated with each specimen.

#### Agathigma

Taxon classificationAnimaliaHymenopteraBraconidae

Sharkey gen. n.

http://zoobank.org/BF65FFF9-3E72-4294-ABB1-F20BD2027EE5

##### Type species.

*Agathigma templei* Sharkey, sp. n.

##### Etymology.

*Aga* (from *Agathis*); *thigma* is Greek for touch, here used as a reference to the reduced 2-segmented palpi. Feminine.

##### Diagnosis.

Body except for fore and mid legs black, hind leg entirely black. Fore wing slightly infuscate in distal half. Antennal sockets not margined with carinae. Interantennal space with a flat triangular elevation that narrows to a short ridge posteriorly approaching the median ocellus. **Temple squared in dorsal view**. **Labial palpus reduced to 2 segments; presumably palpomere 3 is one of the two lost palpomeres**. Notauli depressed and partly or entirely pitted. Scutellar triangle smooth with weak sparse punctures. Ventral margin of hind coxal cavities situated below dorsal margin of metasomal foramen. Pegs on anterior surface of fore tibia absent. Hind trochantellus lacking longitudinal carinae. **Second submarginal cell of fore wing minute, cell about the same diameter as wing veins.** First median tergite almost entirely irregularly striate, lateral longitudinal carina prominent. Second median tergite slightly wider than long and entirely smooth with hints of short striae and some very weak coriarious microsculpture.

##### Distribution and diversity.

Known only from the type specimen collected in Mae Wong National Park, Thailand.

#### Agathigma templei

Taxon classificationAnimaliaHymenopteraBraconidae

Sharkey sp. n.

http://zoobank.org/983A6820-7326-4051-9683-5A35EDD0BE93

##### Etymology.

Named after Jimmy Temple, childhood friend of the first author; the fact that the temples are squared may be coincidental.

**Figure 2.**
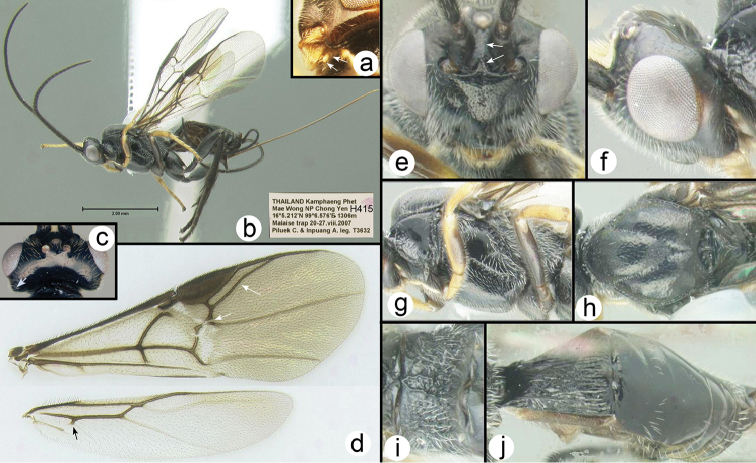
*Agathigma templei* holotype female: **a** labial palpus, arrows indicate the two palpomeres **b** lateral habitus **c** dorsal head **d** wings; arrows from top to bottom indicate: RS vein; minute second submarginal cell; angle in cu-a crossvein of hind wing **e** anterodorsal head, arrows indicate ridge between antennae **f** lateral head **g** lateral mesosoma **h** dorsal mesonotum **i** dorsal propodeum **j** dorsal metasoma.

##### Diagnosis.

Body length 4.7 mm. Ovipositor length/body length ratio = 1.0. Interantennal space with a flat triangular elevation that narrows to a short ridge posteriorly approaching the median ocellus. Antenna with 30 flagellomeres. Labial palp reduced, 2-segmented. Notauli pitted anteriorly, smooth posteriorly where they converge. Scutellar triangle and its posterior surface unusually smooth. Scutellar groove with 1 longitudinal ridge. Fore tibia lacking spines or pegs; mid tibia with 3 pegs; hind tibia with 5 pegs. Basal lobe of tarsal claws large and right-angled, claw only extending slightly beyond apex of lobe. RS vein of fore wing slightly sinuate. Second submarginal cell minute. Hind tibial spurs melanic. Hind tibia entirely melanic. Cu-a crossvein of hind wing bent at point where it is intersected by vein Cub.

##### Specimens examined.

Holotype 2♀♀ (H415): THAILAND, Kamphaeng Phet, Mae Wong NP Chong Yen, 16°5.212'N, 99°6.576'E, 1306 m, Malaise trap, 20–27.viii.2007, Piluek C. & Inpuang A. leg.

##### Distribution.

Known only from the type specimen collected in Mae Wong National Park, Thailand. For a distribution map go to: http://bit.ly/22WV8JD

#### Asperagathis

Taxon classificationAnimaliaHymenopteraBraconidae

Sharkey gen. n.

http://zoobank.org/DF7D4C7E-A82A-4F34-8226-6B850C3F07EC

##### Type species.

*Asperagathis xesta* Sharkey, sp. n.

##### Etymology.

*Asper* is Latin for rough; here it is in reference to the rugose sculpture on the thoracic pleura of members of the genus. Feminine.

##### Diagnosis.

Body predominantly black; head including orbits of eyes black; dorsal apex of pronotum pale yellow or yellowish brown; metasomal terga all black. Fore wing slightly infuscate in apical half or entirely clear/hyaline. Antennal sockets not margined with carinae. Interantennal space with a shallow crest; between the crest and the median ocellus there is a triangular depression flanked by weak smooth carinae. Temple rounded in dorsal view. Third labial palpomere not greatly reduced, about 1/2 as long as apical palpomere. Notauli depressed and entirely sculptured. **Mesoscutum with more rough sculpture than most genera, especially posteriorly near junction of notauli**. Scutellar triangle rugose or with deep sparse punctures. **Sternaulus completely sculptured to epicnemium, metapleuron covered in rough sculpture**. Ventral margin of hind coxal cavities situated below dorsal margin of metasomal foramen. Spines or pegs on anterior surface of fore tibia present or absent. Hind trochantellus lacking longitudinal carinae. Second submarginal cell of fore wing varying from minute, cell about the same diameter as wing veins, to petiolate with petiole slightly longer than cell diameter. First median tergite mostly to about 1/2 irregularly striate, lateral longitudinal carina prominent. Second median tergite slightly wider than long and entirely smooth with some very weak coriarious microsculpture, varying to almost entirely irregularly longitudinally striate, with striae terminating evenly near apex of tergite.

##### Distribution and diversity.

Thailand, but undoubtedly more widespread.

##### Biology.

Unknown.

##### Key to the Thai species of *Asperagathis*

**Table:** 

1	**a.** Metasomal median tergite 2 mostly or entirely smooth. **aa.** Second submarginal cell minute, diameter about equal to thickness of surrounding wing veins	***Asperagathis xesta* Sharkey, sp. n.**
–	**b.** Metasomal median tergite 2 mostly rugosostriate. **bb.** Second submarginal cell normal (wider than below), diameter much wider than thickness of surrounding wing vein	***Asperagathis aspera* Sharkey, sp. n.**

**Asperagathis key,:**
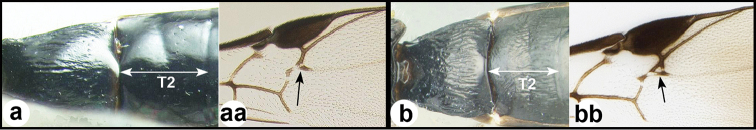
Figure 1.

#### Asperagathis aspera

Taxon classificationAnimaliaHymenopteraBraconidae

Sharkey sp. n.

http://zoobank.org/898E350A-2A6F-4901-913F-7C04054FC4BB

##### Etymology.

*Asper* is Latin for rough and refers to the sculpture of the second metasomal median tergite.

##### Diagnosis.

Body length 7.6 mm; ovipositor length/body length ratio = 1.0. Interantennal space with a flat triangular elevation that narrows to a short ridge posteriorly and then divides into two short carinae that diverge to either side of the median ocellus. Antenna with 38 flagellomeres. 3^rd^ labial (penultimate) palpomere long, about ½ as long as apical palpomere. Scutellar groove with 3-4 longitudinal ridges. Fore tibia with about 9 thickened spines concolorous with normal setae; mid tibia with 7 pegs; hind tibia with 8 pegs.

**Figure 3.**
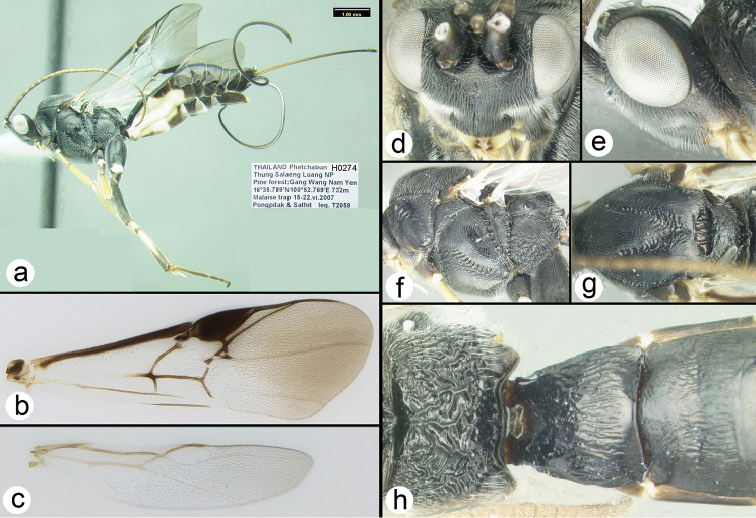
*Asperagathis aspera*, holotype female: **a** lateral habitus **b** fore wing **c** hind wing **d** dorsal head **e** lateral head **f** lateral mesosoma **g** dorsal thorax **h** propodeum and metasomal terga 1-2.

##### Specimens examined.

**Holotype** ♀ (H274): THAILAND, Phetchabun , Thung Salaeng Luang NP, Pine forest; Gang Wang Nam Yen, 16°35.789'N, 100°52.769'E, 732 m, Malaise trap, 15–22.vi.2007, Pongpitak & Sathit leg.

##### Distribution.

Known only from the type specimen collected in Thung Salaeng Luang National Park, Thailand. For a distribution map go to: http://bit.ly/1T5FqXj

#### Asperagathis xesta

Taxon classificationAnimaliaHymenopteraBraconidae

Sharkey sp. n.

http://zoobank.org/A2028B25-6FEF-45D6-980E-A1375A966D5F

##### Etymology.

*Xestos* is Greek for smooth and refers to the smooth second metasomal median tergite.

##### Diagnosis.

Body length 4.5 mm; ovipositor length/body length ratio = 1.1. Interantennal space with a flat triangular elevation that narrows to a short ridge posteriorly and then divides into two short carinae that diverge to either side of the median ocellus. Antenna with 32 flagellomeres. 3^rd^ labial (penultimate) palpomere long, more than ½ as long as apical palpomere. Scutellar groove with 3 longitudinal ridges. Fore tibia lacking thickened spines; mid tibia with 6 pegs; hind tibia with 10 pegs.

**Figure 4.**
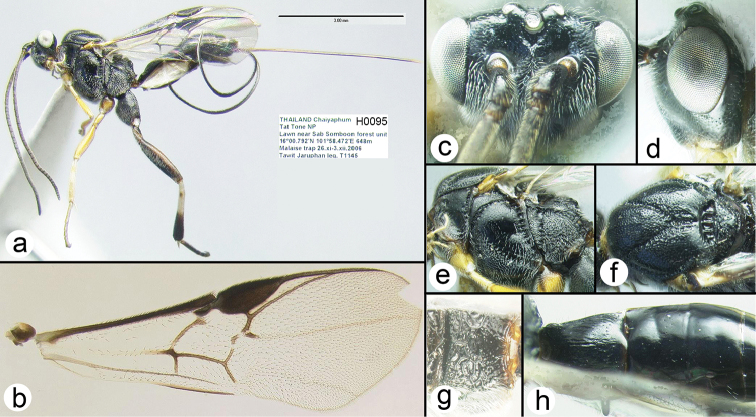
*Asperagathis xesta* Holotype female: **a** lateral habitus **b** fore wing **c** dorsal head **d** lateral head **e** lateral mesosoma **f** dorsal thorax **g** propodeum **h** metasomal terga 1-3.

##### Specimens examined.

**Holotype** ♀ (H095): THAILAND, Chaiyaphum, Tat Tone NP, Lawn near Sab Somboon forest unit, 16°0.792'N, 101°58.472'E, 648m, Malaise trap, 26.xi–3.xii.2006, Tawit Jaruphan leg. **Paratype** ♀ (H1682): Same data as holotype.

##### Distribution.

Known only from the type specimens collected in Tat Tone National Park, Thailand. For a distribution map go to: http://bit.ly/1VPL5H8

#### Chimaeragathis

Taxon classificationAnimaliaHymenopteraBraconidae

Sharkey gen. n.

http://zoobank.org/19BD7E73-FE7C-4947-B3B2-4B2F5C32E41C

##### Type species.

*Chimaeragathis eurysoma* Sharkey, sp. n.

##### Etymology.

Chimaera is a mythological Greek monster with a goat’s body, lion’s head, and serpent’s tail. In this case, the name is a reference to the many diagnostic characters of the genus which are a combination of features each of which diagnoses other agathidine genera, e.g., crest between antennae, fore tibia with thickened spines. Feminine.

##### Diagnosis.

**Metapleuron, scutellum, and all but median cell of propodeum thickly setose**. Head, including orbits of eye, black; mesosoma black; metasoma variable. Fore wing slightly infuscate in apical half or entirely clear/hyaline. Antennal sockets not margined with carinae. Interantennal space with a high crest that is sharply declivous posteriorly; between the crest and the median ocellus there is a triangular depression flanked by weak smooth carinae. Temple rounded in dorsal view. Third labial palpomere small, less than 1/3 length of apical palpomere. Notauli depressed and partly or entirely pitted. **Scutellar triangle rugose**. Ventral margin of hind coxal cavities situated below dorsal margin of metasomal foramen. Pegs on anterior surface of fore tibia present. Hind trochantellus lacking longitudinal carinae. Second submarginal cell of fore wing varying from minute, cell about the same diameter as wing veins, to petiolate with petiole longer than cell diameter. First median tergite partly or mostly irregularly striate to rugosostriate, otherwise smooth; lateral carina present, sometimes weak; median carina present, sometimes weak. Second median tergite wider than long and smooth or mostly smooth with some irregular striae.

##### Distribution and diversity.

Undescribed species are found in other Southeast Asian countries.

##### Biology.

Unknown.

##### Key to Thai species of Chimaeragathis

**Table:** 

1	**a.** Hind femur yellow; hind tibia mostly yellow	***Chimaeragathis lohmani* Sharkey, sp. n.**
–	**b.** Hind femur yellow laterally at mid length, black basally and apically, hind tibia black.	***Chimaeragathis chrysoma* Sharkey, sp. n.**
–	**c.** Hind femur black; hind tibia mostly black with pale patches basally	***Chimaeragathis eurysoma* Sharkey, sp. n.**

**Chimaeragathis key:**
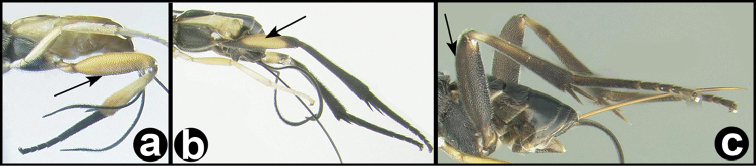
Figure 1.

#### Chimaeragathis chrysoma

Taxon classificationAnimaliaHymenopteraBraconidae

Sharkey sp. n.

http://zoobank.org/46EB8961-A3DE-4951-B077-459C9F7481F5

##### Etymology.

*Chrysoma* is Greek for an object made of gold and is a reference to the gold colored setae on the mesosoma.

##### Diagnosis.

Body length 6.9 mm; ovipositor length/body length ratio = 0.8. Antenna with 42 flagellomeres. Third labial (penultimate) palpomere about 1/3 as long as apical palpomere. Propleuron convex, lacking distinct bump. Scutellar groove with 3 longitudinal ridges. Fore tibia with 2 pegs; mid tibia with 5 pegs; hind tibia with 4 pegs. Basal lobe of tarsal claws large, right-angled; claw extending slightly beyond apex of lobe.

**Figure 5.**
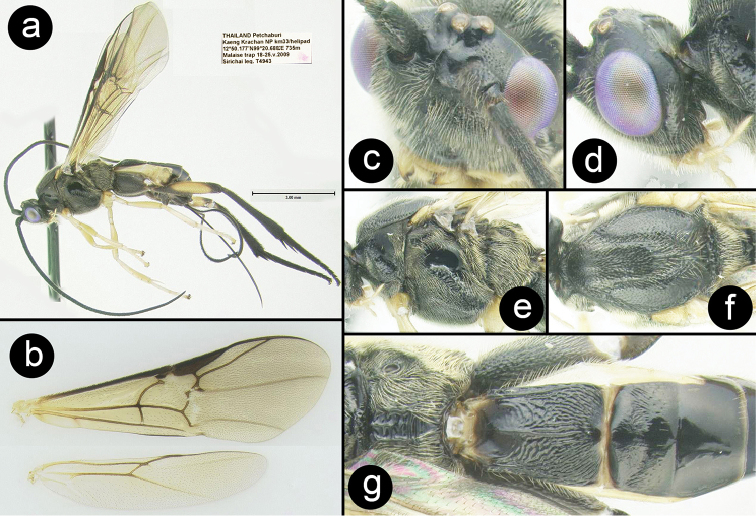
*Chimaeragathis chrysoma* holotype female: **a** lateral habitus **b** wings **c** anterolateral head **d** lateral head **e** lateral mesosoma **f** dorsal thorax **g** propodeum and metasomal terga 1-3.

##### Specimens examined.

**Holotype** ♀ (H710): THAILAND, Petchaburi, Kaeng Krachan NP km33/helipad, 12°50.177'N, 99°20.688'E, 735 m, Malaise trap, 18-25.v.2009, Sirichai leg.

##### Distribution.

Known only from the type specimen collected in Kaeng Krachan National Park, Thailand. For a distribution map go to: http://bit.ly/29nOQlL

#### Chimaeragathis eurysoma

Taxon classificationAnimaliaHymenopteraBraconidae

Sharkey sp. n.

http://zoobank.org/664E801F-8A72-40E1-99A6-48FC848F974B

##### Etymology.

*Eurys* is Greek for wide; *soma* is Greek for body. The species name refers to the wide metasoma of this species.

##### Diagnosis.

Body length 4.8 mm; ovipositor length/body length ratio = 0.7. Antenna with 34 flagellomeres. Third labial (penultimate) palpomere about 1/3 as long as apical palpomere. Propleuron convex, lacking distinct bump. Scutellar groove with 3 longitudinal ridges. Fore tibia with 3 pegs; mid tibia with 4 pegs; hind tibia with 3 pegs. Basal lobe of tarsal claws large, right-angled; claw extending slightly beyond apex of lobe.

**Figure 6.**
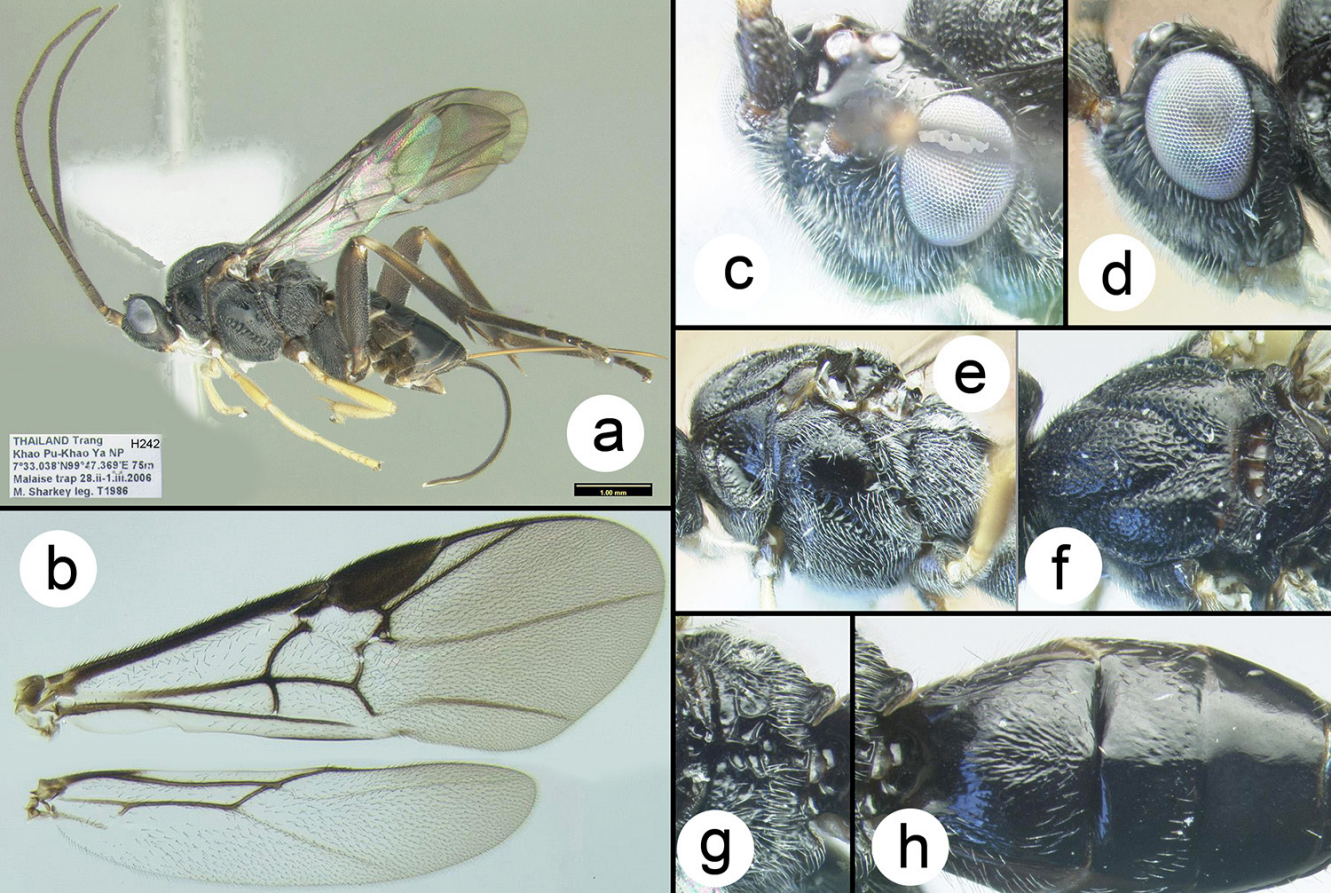
*Chimaeragathis eurysoma*, female paratypes: **a** lateral habitus **b** wings **c** anterolateral head **d** lateral head **e** lateral mesosoma **f** dorsal thorax **g** propodeum **h** metasomal terga 1-3.

##### Specimens examined.

**Holotype** ♀ (H925): THAILAND, Petchaburi, Kaeng Krachan NP, km33/helipad, 12°50.177'N, 99°20.688'E, 735 m, Malaise trap, 25.i–4.ii.2009, Sirichai leg. **Paratypes**: THAILAND: ♀ (H321), Prachuab Khiri Khan, Khao Sam Roi Yot NP Khao Look Glang 12°6.414'N, 99°57.292'E, Malaise trap, 28.ix–5.x.2008, Yai Amnad leg. ♀ (H242), Trang, Khao Pu-Khao Ya NP, 7°33.038'N, 99°47.369'E, 75 m, Malaise trap, 28.ii–1.iii.2006 M Sharkey leg. ♀ (H649), Chanthaburi, Khao Khitchakut NP, nature trail/fern, 12°50.55'N, 102°7.3'E, 50 m, Malaise trap, 1–8.v.2009, Suthida Charoenchai leg. ♀ (H335), Chanthaburi, Khao Khitchakut NP, nature trail/Banyan tree, 12°50.54'N, 102°7.31'E, 90 m, Malaise trap, 1–8.v.2009, Suthida Charoenchai leg. ♀ (H045), Trang, nr. nam Tok Ton Prew Kae Chong, MT, 140 m, 7°33.15'N, 99°47.38'E, 28.i–3.ii.2005 D Lohman. ♀ (H069), Trang, nr. nam Tok Ton Prew Kae Chong, MT, 140 m, 7°33.15'N, 99°47.38'E, 4–11.ii.2005 D Lohman.

##### Distribution.

Known only from the type specimens collected in Thailand. For a distribution map go to: http://bit.ly/1WNrlTX

#### Chimaeragathis lohmani

Taxon classificationAnimaliaHymenopteraBraconidae

Sharkey sp. n.

http://zoobank.org/54EB7934-9C28-47C5-924C-ECD9A8425A41

##### Etymology.

Named after David Lohman, who collected of one of the specimens in the type series and who serviced Malaise traps in Trang Province for many months.

##### Diagnosis.

Body length 6.2 mm; ovipositor length/body length ratio = 0.8. Antenna with 39 flagellomeres. Third labial (penultimate) palpomere about 1/3–1/2 as long as apical palpomere. Propleuron convex, lacking distinct bump. Scutellar groove with 3 longitudinal ridges. Fore tibia with 1 peg; mid tibia with 3 pegs; hind tibia with 4 pegs. Basal lobe of tarsal claws large, right-angled; claw extending slightly beyond apex of lobe.

**Figure 7.**
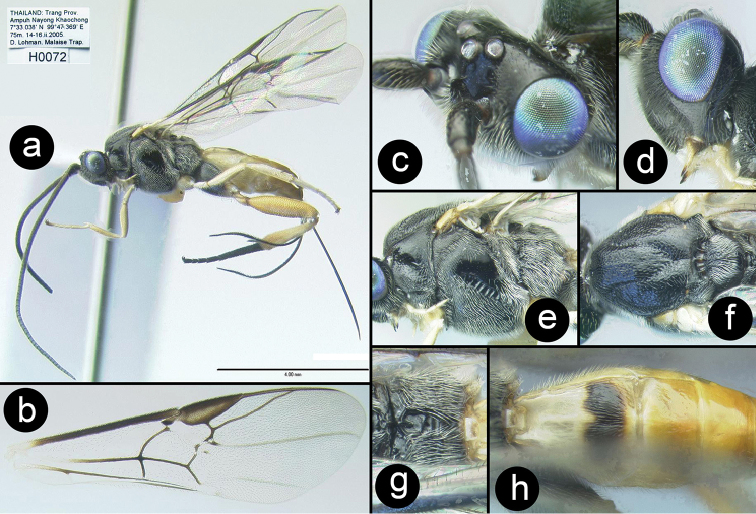
*Chimaeragathis lohmani*. **a** and **c–h** Holotype female **b** fore wing of paratype H412 **c** anterolateral head **d** lateral head **e** lateral mesosoma **f** dorsal thorax **g** propodeum **h** metasomal terga 1-3.

##### Specimens examined.

**Holotype** ♀ (H072), THAILAND, Trang, Ampuh Nayong Khaochong, 7°33.038'N, 99°47.369'E, 75 m, 14–16.ii.2005, Mal. trap D Lohman. **Paratypes**: THAILAND: ♀ (H077), Trang, Khaochong, 7°33.038'N, 99°47.369'E, 75 m, 13.vi.2005, Mal. trap. ♀ (H412), Surat Thani, Khao Sok NP Klong Morg Unit, 8°53.725'N, 98°39.025'E, 87 m, Malaise trap, 10–17.ii.2009, Pongphan leg. Malaysia: 2♀♀ (H5932, H5935), Perlis, Wang Kelian, 6°40'40.94"N, 100°11'23.94"E, 2008, Sharkey and Norliyana.

##### Distribution.

Known only from the type specimens collected in northern Malaysia and southern Thailand. For a distribution map go to: http://bit.ly/1r7TE3x

#### Cymagathis

Taxon classificationAnimaliaHymenopteraBraconidae

Sharkey gen. n.

http://zoobank.org/5B95835D-D202-428C-BFAF-C3D8564B55B1

##### Type species.

*Cymagathis krikoma* Sharkey, sp. n.

##### Etymology.

*Cymato* is Greek for wave; here it is a reference for the uniform, large, wave-like striae on metasomal median tergite 2. Feminine.

##### Diagnosis.

Body predominantly black, mesosoma all black, metasomal terga all black, head black except posterior orbit of eyes partly orange. Fore wing slightly infuscate in apical half. Antennal sockets not margined with carinae. Interantennal space with a flat triangular elevation that narrows to a short ridge posteriorly approaching the median ocellus. Temple rounded in dorsal view. Third labial palpomere not greatly reduced, more than 1/2 as long as apical palpomere. Notauli depressed and partly or entirely pitted. Scutellar triangle with dense punctures or aciculations. Ventral margin of hind coxal cavities situated below dorsal margin of metasomal foramen. Pegs on anterior surface of fore tibia present. Hind trochantellus lacking longitudinal carinae. Second submarginal cell of fore wing minute, cell about the same diameter as wing veins. First median tergite evenly and completely covered in strong striae, lateral carinae strong but partly obscured by sculpture. Second median tergite wider than long. **Second median tergite entirely covered with strong striae that end evenly at apex of tergite; striae forming semicircular pattern anteromedially**.

##### Distribution and diversity.

Known only from the type species in Thailand but probably widespread throughout Southeast Asia.

##### Biology.

Unknown

#### Cymagathis krikoma

Taxon classificationAnimaliaHymenopteraBraconidae

Sharkey sp. n.

http://zoobank.org/78E27E64-BCA3-42D8-B0A9-AF01715ED249

##### Etymology.

*Krikoma* is Greek for ring and refers to the half ring-shaped carina on median tergite two.

##### Diagnosis.

Body length 6.0 mm; ovipositor length/body length ratio = 0.9. Scutellar groove with 3 longitudinal ridges. Fore tibia with 4 thickened melanic spines; mid tibia with 3 pegs; hind tibia with 4 pegs. Flagellomeres rather pale colored. Posterior orbit of eye orange. Sternaulus deeply sculptured and long. Metapleuron rugose over most of surface.

**Figure 8.**
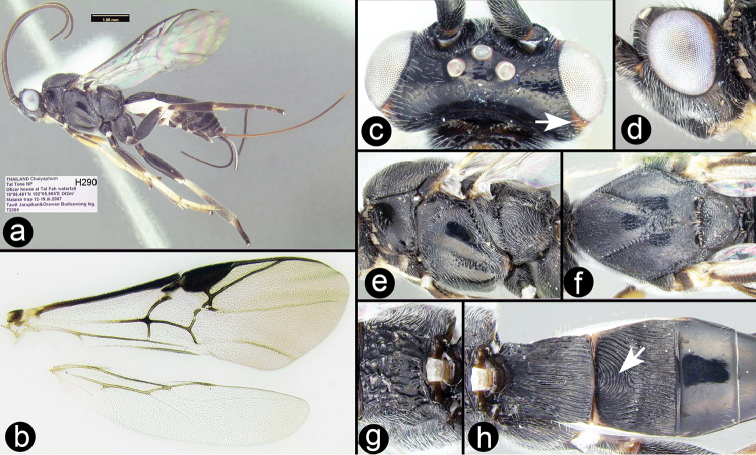
*Cymagathis krikoma* paratype female: **a** lateral habitus **b** wings **c** dorsal head, arrow indicating orange posterior orbit **d** lateral head **e** lateral mesosoma **f** dorsal thorax **g** propodeum **h** metasomal terga 1-3, arrow indicating semicircular carina on median tergite 2.

##### Specimens examined.

**Holotype** ♀ (H276), THAILAND, Chaiyaphum, Tat Tone NP, Water tank at Tat Fah waterfall, 15°56.468'N, 102°5.855'E, 245 m, Malaise trap, 19–26.iii.2007, Tawit Jaruphan & Orawan Budsawong leg. **Paratypes**: THAILAND: ♀ (H290), Chaiyaphum , Tat Tone NP, Officer house at Tat Fah waterfall, 15°56.461'N, 102°5.955'E, 242 m, Malaise trap, 12–19.iii.2007, Tawit Jaruphan & Orawan Budsawong leg. ♀ (H5924), Chaiyaphum, Tat Tone NP, Forest fire Protection station, 16°0.809'N, 102°1.335'E, 195 m, Malaise trap, 3–9.vi.2006, Tawit Jaruphan & Orawan Budsawong leg. ♀ (H2401), Phetchabun, Nam Nao NP Check point, 16°43.695'N, 101°33.797'E, 921 m, Malaise trap, 5–12.v.2007, Leng Janteab leg. ♀ (H483), Mae Hong Son, Namtok Mae Surin NP, Haad Saen, 19°20.857'N, 97°59.123'E, Malaise trap, 27.iv–4.v.2008, Na-maadkam, leg.

##### Distribution.

Known only from the specimens collected in Thailand but *Bassus transtriatus* (Bhat and Gupta) from Philippines may belong here. For a distribution map go to: http://bit.ly/1SWUYfQ

#### Gyragathis

Taxon classificationAnimaliaHymenopteraBraconidae

Achterberg & Long, 2010

##### Diagnosis.

**Antennal sockets margined, completely or at least laterally and medially, with carinae**. Interantennal space with a longitudinal depression bordered by carinae. **Temples squared in dorsal view**. Third labial palpomere minute, barely visible, much smaller than apical palpomere. Notauli depressed and partly or entirely pitted. Scutellar triangle smooth or **rugose**. Ventral margin of hind coxal cavities situated below or in line with dorsal margin of metasomal foramen. Pegs on anterior surface of fore tibia absent. Hind trochantellus lacking longitudinal carinae

##### Distribution and diversity.

There are four species, all of which are restricted to the Oriental region (Taiwan, Philippines, and Viet Nam, Thailand). The three previously described species may be distinguished most easily from *Gyragathis leucosoma* sp. n. by the extensive pale color (yellow to orange) on their mesonota. Achterberg and Long (2010), described the genus *Gyragathis* and the new species *Gyragathis guyi* from Viet Nam. They also transferred three species to the new genus, viz. *Gyragathis angulosa* (Bhat & Gupta, 1977) *Gyragathis parallela* (Chou & Sharkey, 1989) and *Gyragathis daanyuanensis* (Chen & Yang, 2006). The species described here *Gyragathis leucosoma*, is strikingly different from other members of the genus in aspects of sculpture, dimensions, and color, and may belong in its own genus. Molecular data for the described species are lacking to confirm or refute this suspicion.

##### New combinations.

*Gyragathis sabahensis* (Bhat and Gupta), comb. n., from *Agathis*. Contrary to Achterberg and Long (2010) *Bassus daanyuanensis* (Chen & Yang, 2006) is a member of *Therophilus*, *Therophilus daanyuanensis* comb. n.

##### Biology.

Unknown.

#### Gyragathis leucosoma

Taxon classificationAnimaliaHymenopteraBraconidae

Sharkey sp. n.

http://zoobank.org/FECBAA53-5CAD-4F8D-9394-6B6A531A5265

##### Etymology.

*Leucosoma* is Greek for white body. The species name refers to the dense white setae on the metapleuron.

##### Diagnosis.

Body length 6.1 mm; ovipositor length/body length ratio = 0.9. Antenna missing after 28th flagellomere. Third labial (penultimate) palpomere about 1/3 as long as apical palpomere. Propleuron with distinct bump near ventral margin. Scutellar groove with 5 longitudinal ridges. Fore tibia without pegs or thickened spines; mid tibia with 6 pegs; hind tibia with 13 pegs.

**Figure 9.**
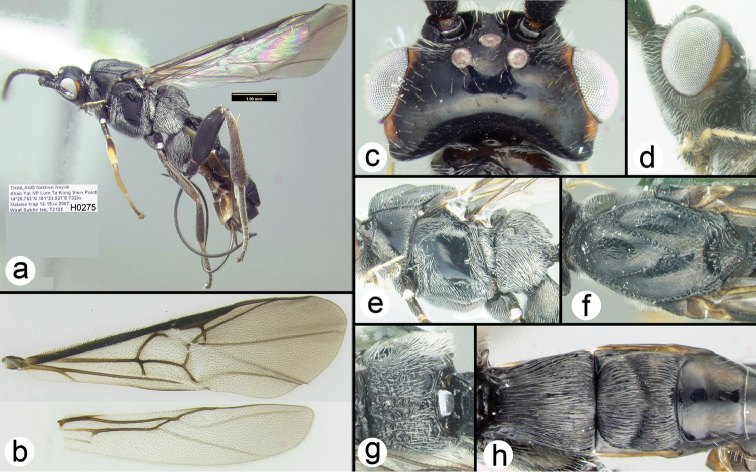
*Gyragathis leucosoma*, female holotype. **a** lateral habitus **b** wings **c** dorsal head **d** lateral head **e** lateral mesosoma **f** dorsal thorax **g** propodeum **h** metasomal terga 1-3.

##### Biology.

Unknown.

##### Specimens examined.

**Holotype** ♀ (H275), THAILAND, Nakhon Nayok, Khao Yai NP, Lum Ta Kong View Point, 14°25.762'N, 101°23.527'E, 732 m, Malaise trap, 12–19.iv.2007, Wirat Sukho leg.

##### Distribution.

Known only from the type specimen collected in Khao Yai National Park, Thailand. For a distribution map go to: http://bit.ly/1SWVgDh

#### Leuroagathis

Taxon classificationAnimaliaHymenopteraBraconidae

Sharkey gen. n.

http://zoobank.org/9B9284AB-0B40-4B80-8A81-851E04E8C174

##### Type species.

*Leuroagathis paulbakeri* Sharkey, sp. n.

##### Etymology.

*Leuros* is Greek for smooth, level, polished and refers to the lack of notauli and smooth metasomal terga. Feminine.

##### Diagnosis.

Head and mesosoma orange and black (head with black in ocellar triangle only); metasomal terga predominantly black with some white. Fore wing slightly infuscate in apical half. Antennal sockets not margined with carinae. Interantennal space with a flat triangular elevation that narrows to a short ridge posteriorly and then divides into two short indistinct carinae that approach the median ocellus. Temple rounded in dorsal view. Third labial palpomere small, less than 1/3 length of apical palpomere. **Notauli completely absent**. Scutellar triangle smooth with weak sparse punctures. Ventral margin of hind coxal cavities situated below dorsal margin of metasomal foramen. Pegs on anterior surface of fore tibia absent. Hind trochantellus lacking longitudinal carinae. Second submarginal cell of fore wing minute, cell about the same diameter as wing veins. First median tergite **smooth, lacking microsculpture and carina**. Second median tergite wider than long. Second median tergite smooth.

##### Distribution and diversity.

Known only from the type species from Thailand. The few Australian Agathidini for which we have COI data do not belong here.

##### Biology.

Unknown.

#### Leuroagathis paulbakeri

Taxon classificationAnimaliaHymenopteraBraconidae

Sharkey sp. n.

http://zoobank.org/5AEB324A-084A-48DF-9FC1-FBDEA27D954A

##### Etymology.

Named in honor of Mr. Paul Baker who obtained the highest mark (100%) in the written exam of Ent. 770 in the fall of 2015.

##### Diagnosis.

Body length 4.5 mm; ovipositor length/body length ratio = 0.8. Interantennal space with a flat triangular elevation that narrows to a short ridge posteriorly and then divides into two short carinae that approach the median ocellus. Antenna with 29 flagellomeres. Third labial (penultimate) palpomere small but easily visible, much smaller than apical palpomere. Scutellar groove with 6 longitudinal ridges. Fore tibia with 7-8 thickened spines; mid tibia with 9 pegs; hind tibia with 12 pegs. First median tergite produced laterally around spiracles. Second median tergite widened apically.

**Figure 10.**
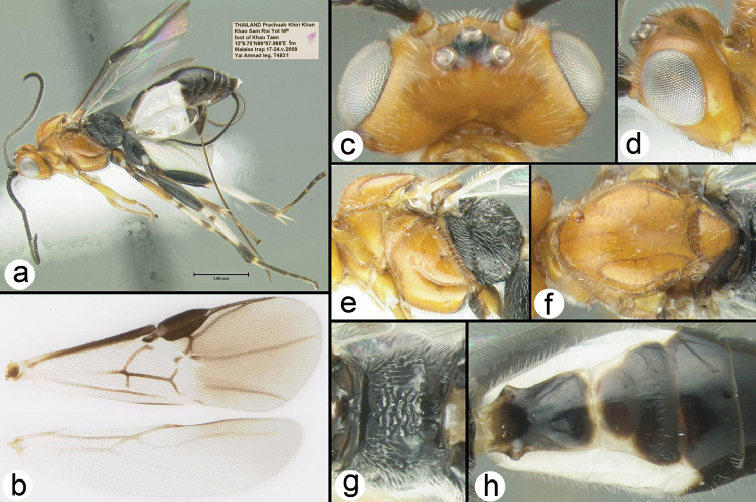
*Leuroagathis paulbakeri* holotype female: **a** lateral habitus **b** wings **c** dorsal head **d** lateral head **e** lateral mesosoma **f** dorsal thorax **g** propodeum **h** metasomal terga 1-3.

##### Specimens examined.

**Holotype** ♀ (H369), THAILAND, Prachuab Khiri Khan, Khao Sam Roi Yot NP, foot of Khao Taen, 12°8.75'N, 99°57.988'E, 1 m, Malaise trap, 17–24.v.2009, Yai Amnad leg.

##### Distribution.

Known only from the type specimen collected in Khao Sam Roi Yot National Park, Thailand. For a distribution map go to: http://bit.ly/29hEQ95

#### Liragathis

Taxon classificationAnimaliaHymenopteraBraconidae

Sharkey gen. n.

http://zoobank.org/70594E06-7AC3-4C4F-96EA-6E95B3F5FE3C

##### Type species.

*Liragathis baonai* Sharkey, sp. n.

##### Etymology.

*Lira* is Latin for ridge, as in the ridge made by a plow in the earth; it is a reference to the median longitudinal ridge on the first metasomal median tergite. Feminine.

##### Diagnosis.

Antennal sockets not margined with carinae. Interantennal space with a flat triangular elevation that narrows to a short ridge posteriorly approaching the median ocellus. Temple rounded in dorsal view. Third labial palpomere, about 1/2 length of apical palpomere. Notauli depressed and partly or entirely pitted. Scutellar triangle smooth or rugose. Dorsal margin of hind coxal cavities situated above ventral-most margin of metasomal foramen. Pegs on anterior surface of fore tibia absent. Hind trochantellus lacking longitudinal carinae. Second submarginal cell of fore wing varying from minute, cell about the same diameter as wing veins, to petiolate with petiole longer than cell diameter. **First median tergite** mostly with irregular striae, lateral and **median carinae strong**. Second median tergite wider than long. Second median tergite from mostly smooth with weak striae restricted to transverse depression, to almost completely striate; in the two species the mostly smooth anteromedial area has transverse or semicircular rugosities, much weaker but otherwise similar to those of *Cymagathis*.

##### Distribution and diversity.

Known from India, Indonesia (Java) and Thailand.

##### Biology.

*Liragathis javana* has been reared from *Etiella zinckenella* (Pyralidae).

##### New combinations.

*Liragathis relativa* (Bhat and Gupta), comb. n. from *Baeognatha*. *Liragathis javana* (Bhat and Gupta), comb. n. from *Baeognatha*.

##### Key to Thai *Liragathis* species

**Table:** 

1	**a.** Mesoscutum mostly or entirely black	2
–	**b.** Mesoscutum mostly or entirely orange	***Liragathis javana* (Bhat & Gupta)**
2	**a.** Superior orbit of eye, between antennal insertion and eye, orange	***Liragathis baonai* Sharkey, sp. n.**
–	**b.** Superior orbit of eye, between antennal insertion and eye, black	***Liragathis damnai* Sharkey, sp. n.**

**Figure 1.**
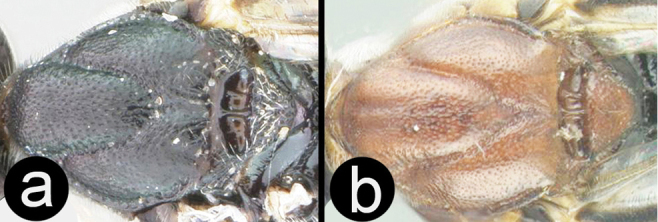
Liragathis key

**Figure 2.**
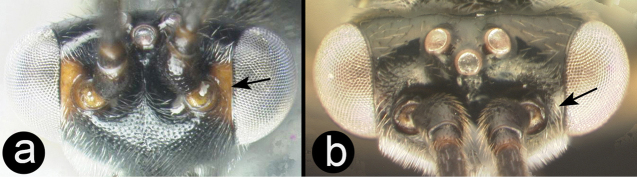
Liragathis key

#### Liragathis baonai

Taxon classificationAnimaliaHymenopteraBraconidae

Sharkey sp. n.

http://zoobank.org/71A70AB7-C1EB-457B-BD59-579A07AD192E

##### Etymology.

*Bao* is Thai for light and *nai* is Thai for eye. The name refers to the pale color of the superior orbit of the eye.

##### Diagnosis.

Body length 6.0 mm; ovipositor length/body length ratio = 1.0. Antenna with 35 flagellomeres. Third labial (penultimate) palpomere about ½ as long as apical palpomere. Scutellar groove with 3 longitudinal ridges. Propodeum rugose and mostly glabrous. Superior orbit of eye orange, posterior orbit also orange. Mesoscutum mostly punctate.

**Figure 11.**
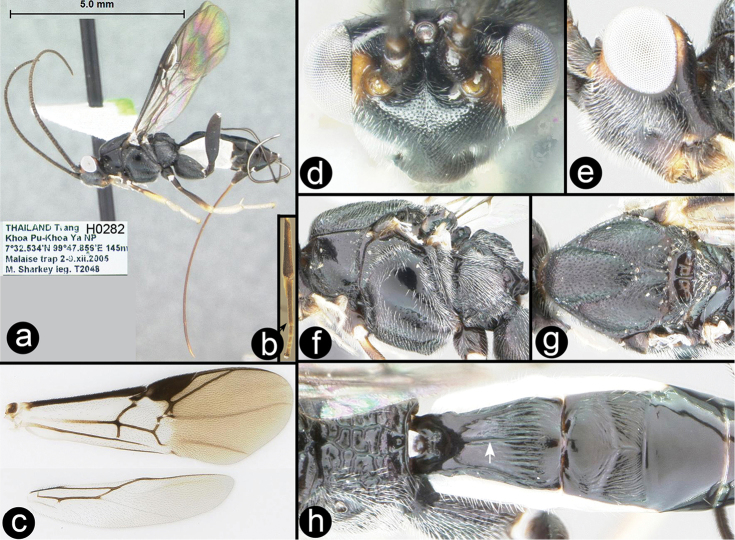
*Liragathis baonai* paratype female: **a** lateral habitus **b** hind tibia and tarsus **c** wings **d** dorsal head **e** lateral head **f** lateral mesosoma **g** dorsal thorax **h** propodeum and metasomal terga 1-3.

##### Specimens examined.

**Holotype** ♀ (H360), THAILAND, Nakhon Si Thammarat, Namtok Yong NP, behind campground lavatory, 8°10.434'N, 99°44.508'E, 80 m, Malaise trap, 9–16.ix.2008, U-prai leg. **Paratypes**: ♀ (H282) THAILAND, Trang, Khao Pu-Khao Ya NP, 7°32.534'N, 99°47.856'E, 145 m, Malaise trap, 2–9.xii.2005 M Sharkey leg. MALAYSIA: 2♀♀ (H5928, H16987), Pahang, Kuala Lompat, 1.ix.1999, 3°41'44.27"N, 102°13'25.42"E, Nor Zaneedarwaty leg. ♀ (H16988), Selangor, Kuala Sawit, 3°11'N, 101°37'E, 22.xi.1999, Nor Zaneedarwaty leg.

##### Distribution.

Known only from the specimens collected in Thailand. For a distribution map go to: http://bit.ly/23QN2Ik

#### Liragathis damnai

Taxon classificationAnimaliaHymenopteraBraconidae

Sharkey sp. n.

http://zoobank.org/DC404004-2052-4A4C-8180-6D012BF3CFE9

##### Etymology.

*Dam* is Thai for black and *nai* is Thai for eye. The name refers to the black color of the superior orbit of the eye.

##### Diagnosis.

Body length 5.3 mm; ovipositor length/body length ratio = 0.7. Antenna with 33 flagellomeres. Third labial (penultimate) palpomere about ½ as long as apical palpomere. Scutellar groove with 3 longitudinal ridges. Fore tibia lacking pegs; mid tibia with 3 pegs; hind tibia with 6 pegs. Fore tibia lacking pegs; mid tibia with 3 pegs; hind tibia with 6–8 pegs. Propodeum rugose but with discernible large areolae as in some *Lytopylus* species. Superior orbit of eye black, posterior orbit orange. Mesoscutum mostly rugose.

**Figure 12.**
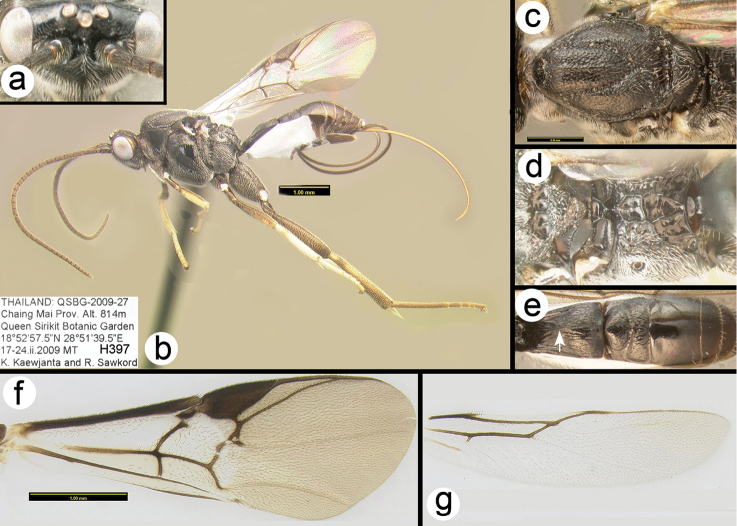
*Liragathis damnai* paratype female: **a** dorsal head **b** lateral habitus **b** hind tibia and tarsus **c** dorsal thorax **d** scutellum and propodeum **e** metasomal terga 1-3 **f** fore wing **g** hind wing.

##### Specimens examined.

**Holotype** ♀ (H468), THAILAND Chiang Mai, Doi Chiang Dao WS, Pha Tang unit, 19°24.978'N, 98°54.886'E, 526 m, Malaise trap, 24–31.iii.2008, Songkran & Apichart leg. **Paratypes**: THAILAND: ♀ (H999), Lampang Chae Son NP, Youthcamp/meeting hall, 18°49.866'N, 99°28.209'E, 476 m, Malaise trap, 15-22.iii.2008 B Kwannui & A. Sukpeng leg. ♀ (H2416), Lampang Chae Son NP Youthcamp, 18°49.826'N, 99°28.256'E, 455 m, Malaise trap, 1–7.iv.2008 B Kwannui & A. Sukpeng leg. ♀ (H397), Chiang Mai, Queen Sirikit Botanic Garden, 18°52'57.5"N, 98°51'35.5"E, 17–24.ii.2009, MT K Kaewjanta & R. Sawkord leg.

##### Distribution.

Known only from the specimens collected in Thailand. For a distribution map go to: http://bit.ly/22WZjoH

#### Liragathis javana

Taxon classificationAnimaliaHymenopteraBraconidae

(Bhat and Gupta)

Baeognatha javana Bhat & Gupta, 1977.

##### Diagnosis.

Body length 6.0 mm; ovipositor length/body length ratio = 0.8. Antenna with 33 flagellomeres. Third labial (penultimate) palpomere about ½ as long as apical palpomere. Scutellar groove with 3 longitudinal ridges. Fore tibia lacking pegs; mid tibia with 4 pegs; hind tibia with 5 pegs. Posterior orbit of eye orange. Mesoscutum, scutellum, pronotum and part of mesopleuron orange. Second median tergite wide, about two times wider than long. Similar to *Liragathis relativa* (Bhat and Gupta). Second submarginal cell of *Liragathis javana* much larger.

**Figure 13.**
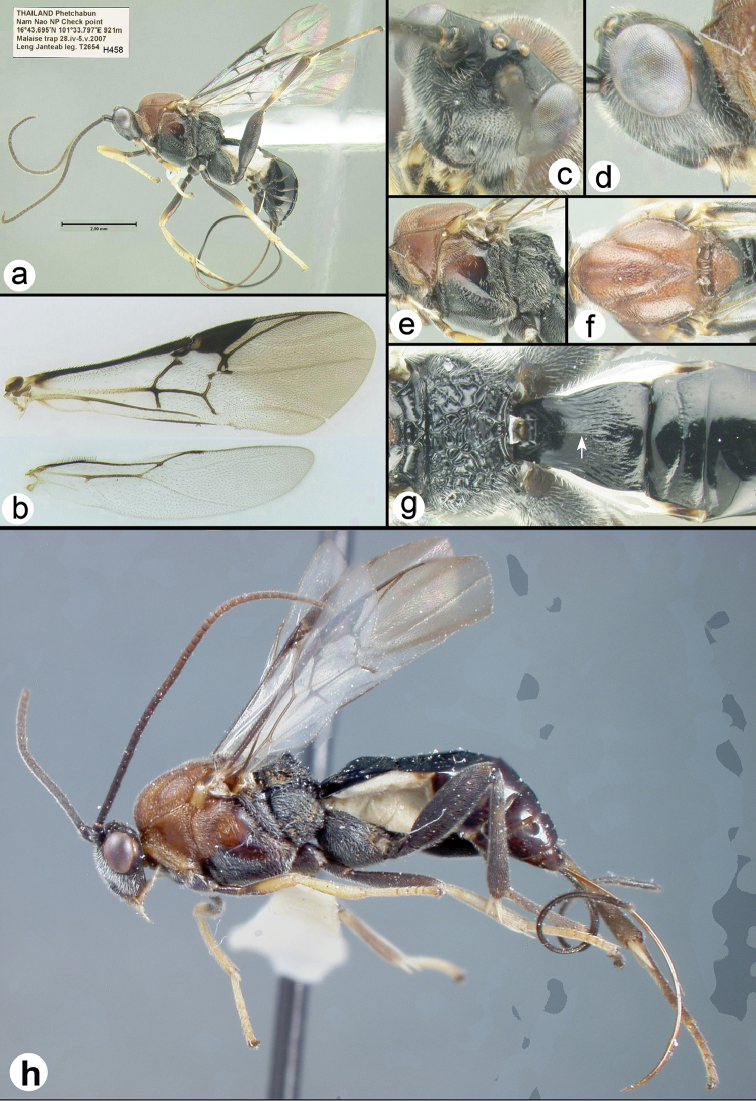
*Liragathis javana* female: **a** lateral habitus **b** wings **c** dorsal head **d** lateral head **e** lateral mesosoma **f** dorsal thorax **g** propodeum and metasomal terga 1-3 **h** holotype lateral habitus.

##### Specimens examined.

**Holotype** ♀, Indonesia, Java, Bogor (=Buitenzorg), 15.ix.1956, ex. Etiella zinckenella M Satarchi, USNM, examined). **Other**: Indonesia: 5♀♀, 2♂♂ (H16989 - H16992), Central Java, Tepus, 6°49'S, 110°53'E, 7.v.1990, host *Etiella* sp. [Pyralidae], coll. G.C. Luther (EMEC).Thailand: ♀ (H628), Phetchabun, Nam Nao NP check point, 16°43.687'N, 101°33.754'E, 924 m, Malaise trap, 5–12.v.2007, Noopean Hongyothi leg. ♀ (H458), Phetchabun, Nam Nao NP check point, 16°43.695'N, 101°33.797'E, 921 m, Malaise trap, 28.iv–5.v.2007, Leng Janteab leg. ♀ (H2406), Phetchabun, Nam Nao NP check point 16°43.695'N, 101°33.797'E, 921 m, Malaise trap, 5–12.v.2007, Leng Janteab leg. ♀ (H419), Kanchanaburi, Khuean Srinagarindra NP, Tha Thung-na/Chong Kraborg, 14°29.972'N, 98°53.035'E, 210 m, Malaise trap, 19–26.iii.2009, Boonnam & Phumarin leg. ♀ (H366), Kanchanaburi, Khuean Srinagarindra NP, Tha Thung-na/Chong Kraborg, 14°29.972'N, 98°53.035'E, 210 m, Malaise trap, 26.iii–2.iv.2009, Boonnam & Phumarin leg.

##### Distribution.

Known only from the specimens collected in Thailand and Indonesia. For a distribution map go to: http://bit.ly/2ajVCca

#### Scabagathis

Taxon classificationAnimaliaHymenopteraBraconidae

Sharkey gen. n.

http://zoobank.org/AF5DB06A-7A3F-466D-85A0-ABA92D2D2F85

##### Type species.

*Scabagathis emilynadeauae* Sharkey, sp. n.

##### Etymology.

*Scaber* is Latin for rough, scabby, mangy; here it refers to the rough (rugose) sculpture on the vertex of the head. Feminine.

##### Diagnosis.

**Vertex of head with rugose sculpture**. Head and mesosoma both black and orange; metasomal terga mostly black; base of first median tergite whitish; basal half of second median tergite whitish yellow. Fore wing **hyaline, not more infuscate in distal half**. Antennal sockets not margined with carinae. Interantennal space with a flat triangular elevation that narrows to a short ridge posteriorly. Temple rounded in dorsal view. **Third labial palpomere absent, palpus 3-segmented**. Notauli depressed and partly or entirely pitted. Scutellar triangle **rugose**. Dorsal margin of hind coxal cavities situated above ventral-most margin of metasomal foramen. Pegs on anterior surface of fore tibia absent. Hind trochantellus lacking longitudinal carinae. Second submarginal cell of fore wing minute, cell about the same diameter as wing veins. First median tergite entirely, finely, irregularly striate; lateral carina weak. Second median tergite **longer than wide**. Second median tergite entirely, finely, irregularly striate, with striae ending evenly near apex of tergite.

##### Distribution and diversity.

Known only from the type species from Thailand.

##### Biology.

Unknown.

#### Scabagathis emilynadeauae

Taxon classificationAnimaliaHymenopteraBraconidae

Sharkey sp. n.

http://zoobank.org/61C5A34B-8C38-4DEA-ADC5-016B42790918

##### Etymology.

Named in honor of Ms. Emily Nadeau who obtained the highest mark in the weekly quizzes of Ent. 770 in the fall of 2015.

##### Diagnosis.

Body length 5.1 mm; ovipositor length/body length ratio = 0.6. Antenna with 31 flagellomeres. Scutellar groove with 3 longitudinal ridges. Fore tibia without thickened spines or pegs; mid tibia with 2 pegs; hind tibia missing. First median tergite whitish at extreme base. Second median tergite whitish in basal half.

**Figure 14.**
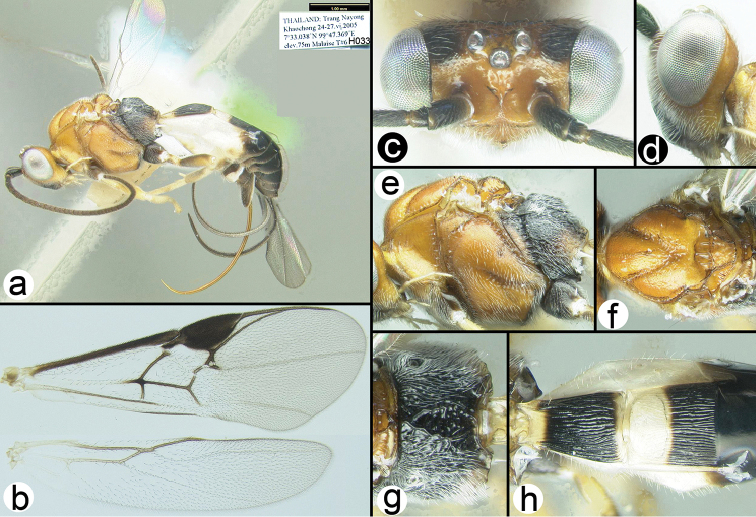
*Scabagathis emilynadeauae* holotype female: **a** lateral habitus **b** wings **c** dorsal head **d** lateral head **e** lateral mesosoma **f** dorsal thorax **g** propodeum **h** metasomal terga 1-3.

##### Specimens examined.

**Holotype** ♀ (H033), Thailand, Trang, Nayong, Khaochong, 24–27.vi.2005, 7°33.038'N, 99°47.369'E, 75 m, Malaise trap.

##### Distribution.

Known only from the type specimen collected in Thailand. For a distribution map go to: http://bit.ly/29kPFrZ

#### Trochantagathis

Taxon classificationAnimaliaHymenopteraBraconidae

Sharkey gen. n.

http://zoobank.org/0F3C1E89-8BBF-4E66-BEAC-352EDA3AF1BB

##### Type species.

*Baeognatha marshi* Bhat & Gupta, 1977

##### Etymology.

*Trochanter* comes from the Greek *trochalos* meaning running; here it is a reference the pair of ridges on the hind trochantellus which are diagnostic for the genus. Feminine.

##### Diagnosis.

Head (including posterior orbit of eye) and mesosoma black; metasomal tergites black or black and pale yellow. Fore wing hyaline, not infuscate in distal half. Antennal sockets not margined with carinae. Interantennal space with a flat triangular elevation that narrows to a short ridge posteriorly and then divides into two short indistinct carinae that approach the lateral margins of the median ocellus. Temple rounded in dorsal view. 3^rd^ labial palpomere minute, barely visible, much smaller than apical palpomere. Notauli depressed and partly or entirely pitted. Scutellar triangle **rugose**. Dorsal margin of hind coxal cavities situated above ventral-most margin of metasomal foramen. Pegs on anterior surface of fore tibia present. **Hind trochantellus with pair of longitudinal carinae**. Second submarginal cell of fore wing varying from about the same diameter as vein Rs about 3x that diameter. First median tergite usually entirely striate, sometimes partly smooth, especially basally; lateral and medial carinae strong. Second median tergite wider than long and varying from completely and smoothly striate, to mostly smooth with weak smooth striae; semicircular pattern of striae usually present anteromedially.

##### Distribution and diversity.

Known from Vietnam, Thailand and Malaysia, but undoubtedly more widespread in the Oriental Region. Based on the analysis of sequence data presented in Figure 1 there are three species of *Trochantagathis* from Thailand. The females of these species are very similar whereas the males appear to be quite different from one another. Males of the more melanic species are almost entirely melanic whereas the males of the other species are only slightly more melanic than their female conspecifics. With the limited molecular data at hand, the sexual dimorphism, and the similarity of the females of the three putative species, it is not possible to tell with confidence which species, if any, corresponds with the type of *Trochanter marshi*. Therefore, we choose not to describe the two or three new species at this time. The specimens from Vietnam placed in *Therophilus marshi*, Achterberg and Long 2010 need verification. They match well with the type except for minor color differences, but so too do the three Thai species. The images of Figure 17 are of a congeneric (and perhaps conspecific) male and female (specimens H799 and H965). These images present better illustrations of the generic characters discussed above as well as the color sexual dimorphism.

##### Biology.

Unknown.

##### New combinations.

*Trochantagathis marshi* (Bhat and Gupta), comb. n., from *Baeognatha*.

#### Trochantagathis marshi

Taxon classificationAnimaliaHymenopteraBraconidae

(Bhat and Gupta) comb. n.

Baeognatha marshi Bhat & Gupta 1977
Therophilus marshi , Achterberg & Long, 2010

##### Diagnosis.

Body length 5.6 mm; ovipositor length/body length ratio = 0.7. Antennae broken (37–38 flagellomeres in Thai congenerics). Third labial (penultimate) palpomere about 1/5 as long as apical palpomere. Scutellar groove with 3 longitudinal ridges. Fore tibia with 2 pegs; mid tibia with 5 pegs; hind tibia with 5 pegs.

**Figure 15.**
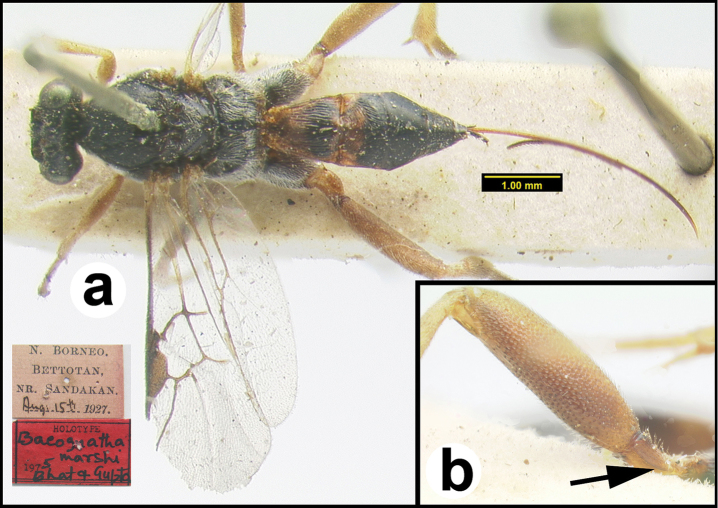
*Trochantagathis marshi* holotype female: **a** dorsal habitus **b** hind femur showing one of the two ridges on the trochantellus.

**Figure 16.**
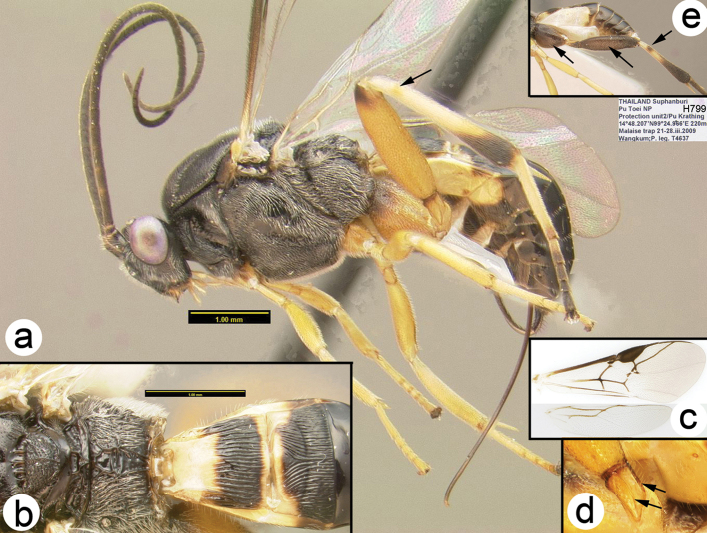
*Trochantagathis marshi*? female: **a** lateral habitus **b** propodeum and metasomal terga 1-2 **c** fore wing **d** detail of ridges on trochantellus of hind leg **e** lateral view of hind leg and metasoma of male (H965); note melanic color of hind coxa and femur.

##### Specimens examined.

**Holotype** ♀, Malaysia, Sabah, Bettotan nr. Sandakan, 15.viii.1927 (FSCA).

##### Distribution.

Malaysia, Vietnam and Thailand. For a distribution map of the Thai specimens go to: http://bit.ly/1VK7I0a

#### Xanthagathis

Taxon classificationAnimaliaHymenopteraBraconidae

Sharkey gen. n.

http://zoobank.org/0F3C1E89-8BBF-4E66-BEAC-352EDA3AF1BB

##### Type species.

*Therophilus mellisoma* Achterberg & Long, 2010.

##### Etymology.

*Xantho* is Greek for yellow and is a reference to the predominantly yellow color of the known species. Feminine.

##### Diagnosis.

**Head yellow, mesosoma and metasoma predominantly yellow, with or without melanic areas. Fore wing hyaline.** Antennal sockets not margined with carinae. Interantennal space with a flat triangular elevation, with a weak shallow ridge posteriorly not as elevated as the triangular elevation. Temple rounded in dorsal view. Third labial palpomere minute, barely visible, much smaller than apical palpomere. Notauli depressed and partly or entirely pitted. Scutellar triangle smooth with weak sparse punctures. Dorsal margin of hind coxal cavities situated above ventral-most margin of metasomal foramen. Pegs on anterior surface of fore tibia absent. Hind trochantellus lacking longitudinal carinae. Second submarginal cell of fore wing minute, cell about the same diameter as wing veins. First median tergite entirely, finely, irregularly striate; lateral carina weak. Second median tergite wider than long. **Second median tergite smooth**.

##### Distribution and diversity.

Viet Nam and Thailand. See below for the distribution of the Thai specimens.

##### Biology.

Unknown.

#### Xanthagathis mellisoma

Taxon classificationAnimaliaHymenopteraBraconidae

(Achterberg and Long) comb. n.

Therophilus mellisoma Achterberg & Long, 2010

##### Diagnosis.

**Figure 17.**
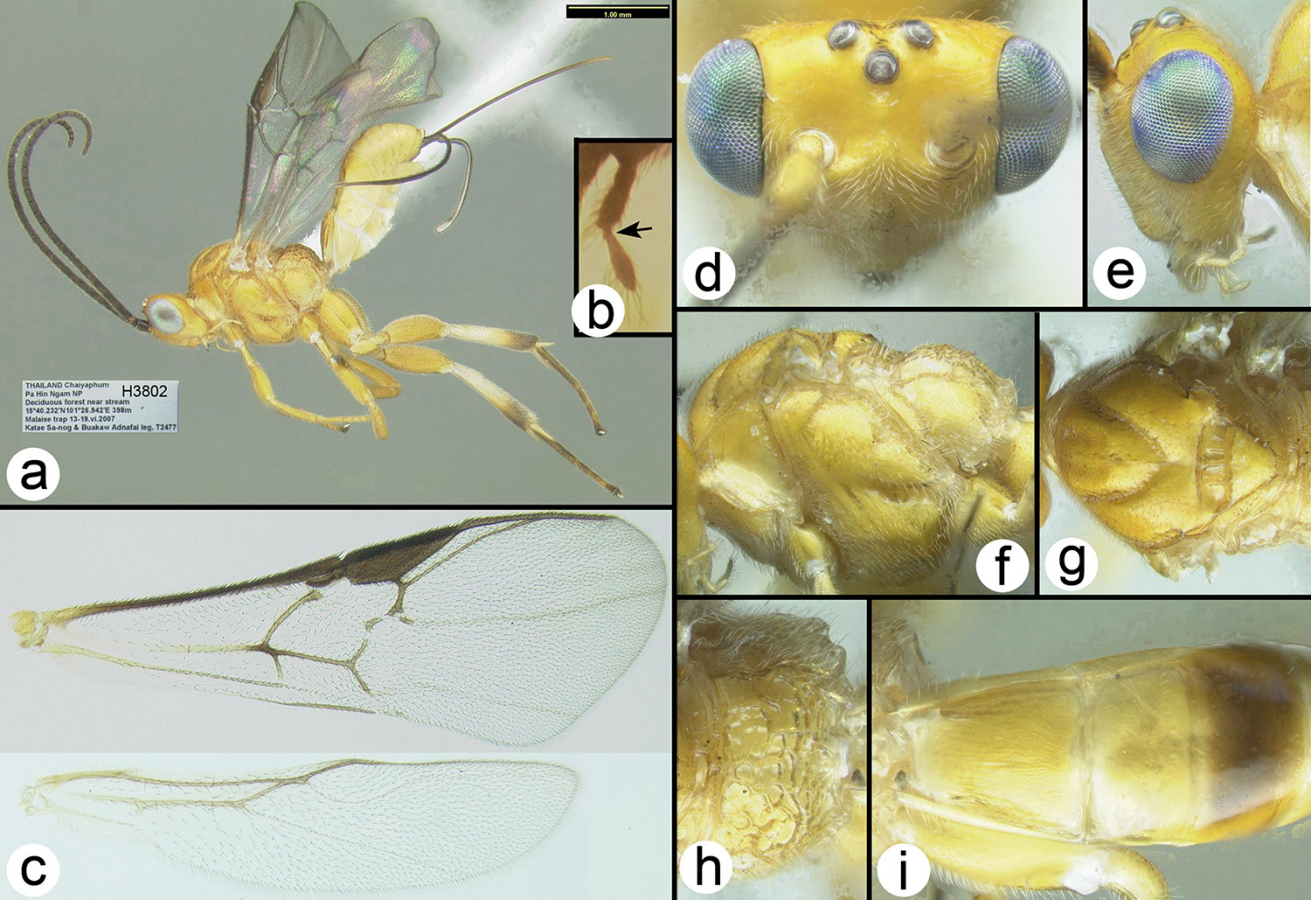
*Xanthagathis mellisoma*, female: **a** lateral habitus **b** labial palp, arrow indicates minute third palpomere **c** wings **d** dorsal head **e** lateral head **f** lateral mesosoma **g** dorsal thorax **h** propodeum **i** metasomal terga 1-3.

Body length 3.7 mm; ovipositor length/body length ratio = 0.64. Interantennal space with a flat triangular elevation, with a weak shallow ridge posteriorly not as elevated as the triangular elevation. Antenna with 27 flagellomeres. Third labial palpomere reduced, barely visible, much smaller than apical palpomere. Notauli pitted throughout. Scutellar groove with 3 longitudinal ridges. Fore tibia lacking spines; mid tibia with 6 pegs; hind tibia with 5 pegs. The Thai specimen in Figure 17 differs from the holotype in the lack of a melanic patch distally on hind femur.

##### Variation.

Color usually entirely xanthic (yellow) except for brown as follows: most wing veins including stigma, antenna, hind tarsus and apex of hind tibia. Some specimens are more melanic with brown color extending to propodeum, most of hind leg and parts of most terga.

##### Distribution.

For a distribution map of the Thai specimens go to: http://bit.ly/1SWVASF

#### Zosteragathis

Taxon classificationAnimaliaHymenopteraBraconidae

Sharkey gen. n.

http://zoobank.org/89E3D8D8-60B7-419A-8129-9D49A0D21EAD

##### Type species.

*Zosteragathis samensis* Sharkey, sp. n.

##### Etymology.

*Zoster* is Greek for belt; here it is a reference to the white band present on the second metasomal median tergite of most species. Feminine.

##### Diagnosis.

Fore wing hyaline, not infuscate in distal half. Antennal sockets not margined with carinae. Temple rounded in dorsal view. Notauli depressed and partly or entirely pitted. Dorsal margin of hind coxal cavities situated above ventral-most margin of metasomal foramen. Pegs on anterior surface of fore tibia absent. Hind trochantellus lacking longitudinal carinae. Second submarginal cell of fore wing petiolate, small to minute. First median tergite entirely, finely, irregularly striate; lateral carina weak. Second median tergite more than 2x longer than wide. **Second median tergite usually entirely, finely, irregularly, striate with striae ending evenly near apex of tergite, rarely striae partly absent. Some species have reduced striae on second median tergite and are recognized by the lack of apomorphic structures that distinguish other closely related genera, e.g., claws not simple, interantennal space without a sharply declivous keel, first median tergite without prominent lateral carina or medial carina, fore tarsus without spines or pegs.**

##### Distribution and diversity.

Australian, Ethiopian, Oceania, Oriental, and eastern Palearctic regions.

##### Biology.

Hosts are unknown for all Thai species; however, there are records for three extra-Thai species. These appear to suggest that the host range is wide. The records are: *Zosteragathis coryphe* was reared from *Phycodes radiata* (Sesioidea: Brachodidae) (Nixon 1950). *Zosteragathis robusta* (Achterberg and Long) from Vietnam was reared from “*Omiodes indicata* (Lepidoptera: Pyralidae: Pyraustinae) on soybean (*Glycine max* (Linnaeus)), according to the label data”, (Achterberg and Long 2010). *Zosteragathis festiva* (Muesebeck) was reared from *Grapholitha molesta*, the oriental fruit moth, (Tortricoidea: Tortricidae). Many other Lepidoptera from a wide range of families are listed by Yu et al. (2012) as hosts of *Zosteragathis festiva*, e.g., Blastobasidae, Carposinidae, Gelechiidae, Noctuidae, and Pyralidae.

### New species combinations

Below is a list of all new combinations that I am aware of. Since the limits, and even the monophyly, of *Zosteragathis* are uncertain the list will undoubtedly change in the future.

*Zosteragathis annulus* (Chou & Sharkey, 1989), **comb. n.** from *Bassus*

*Zosteragathis asper* (Chou & Sharkey, 1989), **comb. n.** from *Bassus*

*Zosteragathis conformis* (Bhat & Gupta, 1977), **comb. n.** from *Agathis*

*Zosteragathis contrasta* (Achterberg & Long, 2010), **comb. n.** from *Therophilus*

*Zosteragathis coryphe* (Nixon, 1950), **comb. n.** from *Agathis*

*Zosteragathis depressa* (Chou & Sharkey, 1989), **comb. n.** from *Bassus*

*Zosteragathis dravida* (Bhat & Gupta, 1977), **comb. n.** from *Agathis*

*Zosteragathis elongator* (Achterberg & Long, 2010), **comb. n.** from *Therophilus*

*Zosteragathis festiva* (Muesebeck, 1953), **comb. n.** from *Agathis*

*Zosteragathis festivoides* (Sharkey, 1996), **comb. n.** from *Bassus*

*Zosteragathis fujianicus* (Chen & Yang, 2006), **comb. n.** from *Bassus*

*Zosteragathis gracilis* (Bhat & Gupta, 1977), **comb. n.** from *Agathis*

*Zosteragathis lienhuachihensis* (Chou & Sharkey, 1989), **comb. n.** from *Bassus*

*Zosteragathis lini* (Chou & Sharkey, 1989), **comb. n.** from *Bassus*

*Zosteragathis masoni* (Bhat & Gupta, 1977), **comb. n.** from *Agathis*

*Zosteragathis nigrolineatus* (Achterberg & Long, 2010), **comb. n.** from *Therophilus*

*Zosteragathis nuichuaensis* (Achterberg & Long, 2010), **comb. n.** from *Therophilus*

*Zosteragathis oranae* (Watanabe, 1970), (syn. of *Zosteragathis festiva*, syn. by Sharkey, 1996), **comb. n.** from *Agathis*

*Zosteragathis parasper* (Achterberg & Long, 2010), **comb. n.** from *Therophilus*

*Zosteragathis punctiscutum* (Achterberg & Long, 2010), **comb. n.** from *Therophilus*

*Zosteragathis robusta* (Achterberg & Long, 2010), **comb. n.** from *Therophilus*

*Zosteragathis scutellatus* (Achterberg & Long, 2010), **comb. n.** from *Therophilus*

*Zosteragathis sungkangensis* (Chou & Sharkey, 1989), **comb. n.** from *Bassus*

*Zosteragathis tanycoleosus* (Chen & Yang, 2006), **comb. n.** from *Bassus*

#### Zosteragathis samensis

Taxon classificationAnimaliaHymenopteraBraconidae

Sharkey sp. n.

http://zoobank.org/36F4AC69-A720-4648-9159-13AF0ECDDE89

##### Etymology.

Named after the type locality Khao Sam Roi Yot National Park.

##### Diagnosis.

Fore coxa yellow. Hind femur black. Second median tergite mostly pale in anterior half and mostly melanic in posterior half. Scutellum sculpture smooth with punctures. Second median tergite dimensions as wide as long or wider.

**Figure 18.**
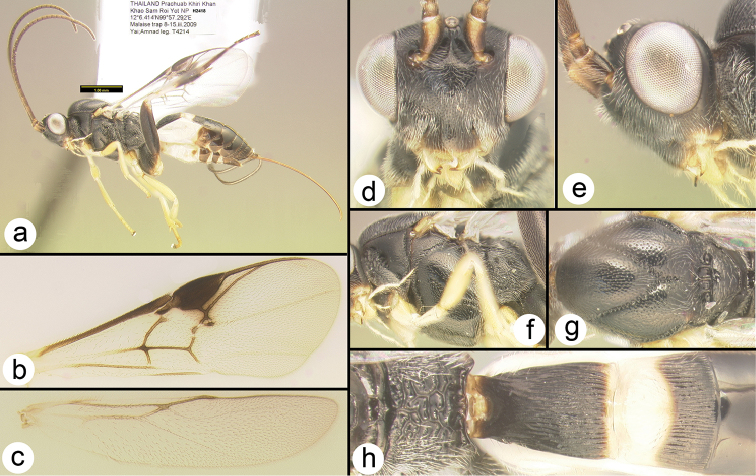
*Zosteragathis samensis*: **a** lateral habitus **b** fore wing **c** hind wing **d** anterior head **e** lateral head **f** lateral mesosoma **g** dorsal mesoscutum **h** propodeum and median tergites 1-3.

##### Description.

Body length 5.4 mm. Ovipositor length 3.3 mm. Ovipositor 0.6 × body length. Number of flagellomeres 31. Notauli sculpture not significantly wider posteriorly. Scutellum smooth with punctures. Mid tibia with 3 apical and 2 preapical spines. Hind tibia with 8 spines/pegs. Second submarginal cell diameter small, smaller than pedicel length, but larger than pedicel width

Wing hyaline with an infuscate patch posterior to stigma. Second median tergite 0.9 × longer than wide. Second median tergite entirely striate, striae weak anteromedially where they converge medially. **Color**: head black except gena yellow; mesosoma black; fore and mid coxa yellow; posterior margin of first median tergite yellow; anterior half of second median tergite yellow.

##### Material examined.

**Holotype**: ♀ (H2418): THAILAND, Prachuab Khiri Khan, Khao Sam Roi Yot NP, Khao Look Glang, 12.107°N, 99.955°E, Malaise trap, 8-15.iii.2009 (H2418), Yai Amnad. **Paratypes**: All ♀: THAILAND, Prachuab Khiri Khan, Khao Sam Roi Yot NP, foot of Khao Taen, 12.146°N, 99.966°E, 1 m elev., Malaise trap, 3–10.v.2009 (H638, H968), Yai Amnad; Prachuab Khiri Khan, Khao Sam Roi Yot NP, 30 m, N/protection unit4, 12.268°N, 99.944°E, 1 m elev., Malaise trap, 3–10.v.2009 (H973, T4824) 24-31.v.2009 (H490), Yai Amnad; Lampang, Chae Son NP, Youthcamp, 18.83°N, 99.471°E, 455 m elev., Malaise trap, 1–7.iv.2008 (H901) B Kwannui & A. Sukpeng; Mae Hong Son, Namtok Mae Surin NP, Haad Saen, 19.348°N, 97.985°E, Malaise trap, 27.iv–4.v.2008 (H481), Na-maadkam, M; Prachuab Khiri Khan, Khao Sam Roi Yot NP, Saline wetland/Pa Gwad/N, 12.153°N, 99.972°E, Malaise trap, 15–22.iii.2009 (H670), Yai Amnad.

##### Distribution.

Known only from the specimens collected in Thailand. For a distribution map go to: http://bit.ly/1MPrTqu

## Supplementary Material

XML Treatment for Agathigma

XML Treatment for Agathigma templei

XML Treatment for Asperagathis

XML Treatment for Asperagathis aspera

XML Treatment for Asperagathis xesta

XML Treatment for Chimaeragathis

XML Treatment for Chimaeragathis chrysoma

XML Treatment for Chimaeragathis eurysoma

XML Treatment for Chimaeragathis lohmani

XML Treatment for Cymagathis

XML Treatment for Cymagathis krikoma

XML Treatment for Gyragathis

XML Treatment for Gyragathis leucosoma

XML Treatment for Leuroagathis

XML Treatment for Leuroagathis paulbakeri

XML Treatment for Liragathis

XML Treatment for Liragathis baonai

XML Treatment for Liragathis damnai

XML Treatment for Liragathis javana

XML Treatment for Scabagathis

XML Treatment for Scabagathis emilynadeauae

XML Treatment for Trochantagathis

XML Treatment for Trochantagathis marshi

XML Treatment for Xanthagathis

XML Treatment for Xanthagathis mellisoma

XML Treatment for Zosteragathis

XML Treatment for Zosteragathis samensis

## Appendix I

**Table:** Specimens used in the phylogenetic analyses, including specimen numbers, GenBank and BOLD accession numbers and rough geographical information.

Taxon name	Specimen number	Country: Region	Type status	COI	28S
*Aerophilus abdominalis*	H1313	USA: KY		ATRMK294-11	KP943685
*Aerophilus malus*	H1484	USA: WV	holotype	ATRMK309-11	KP943693
*Aerophilus rayfisheri*	H1212	USA: KY	holotype	ATRMK278-11	KP943675
*Agathacrista depressifera*	H002	Thailand: Phetchabun		KP943596	KC556782
*Agathacrista krataei*	H268	Thailand: Kalasin	holotype	KP943614	KC556781
*Agathacrista sailomi*	H013	Thailand: Chiang Mai	holotype	KX431796	KC556780
*Agathacrista winloni*	H502	Thailand: Phetchabun	holotype	ATRMK218-11	KC771135
*Agathigma templei*	H415	Thailand: Kamphaeng	holotype	ATRMK211-11	KX431753
*Alabagrus maculipes*	H6020	Mexico: Jalisco		ATRMK370-11	KP943698
*Asperagathis aspera*	H274	Thailand: Phetchabun	holotype	KX431797	KX431706
*Asperagathis xesta*	H095	Thailand: Chaiyaphum	holotype	KX431798	KX431707
*Bassus albifasciatus*	H014	Thailand: Sakon Nakhon		------	KX431714
*Bassus albifasciatus*	H027	Thailand: Trang		KX431800	KX431716
*Bassus albifasciatus*	H032	Thailand: Trang		KX431799	KX431715
*Bassus albifasciatus*	H085	Thailand: Trang		KX431801	KX431719
*Bassus albifasciatus*	H343	Thailand: Chiang Mai		------	KX431718
*Bassus albifasciatus*	H377	Thailand: Nakhon Si Thammarat		------	KX431717
*Bassus alboapicalis*	H021	Thailand: Trang	paratype	KX431821	KX431767
*Bassus alboapicalis*	H022	Thailand: Trang	paratype	KX431819	KX431764
*Bassus alboapicalis*	H081	Thailand: Trang	paratype	KX431817	KX431762
*Bassus alboapicalis*	H269	Thailand: Trang	holotype	KX431820	KX431766
*Bassus alboapicalis*	H270	Thailand: Trang	paratype	KX431818	KX431763
*Bassus alboapicalis*	H307	Thailand: Surat Thani	paratype	ATRMK195-11	KX431765
*Bassus alboapicalis*	H410	Thailand: Nakhon Si Thammarat	paratype	------	KX431761
*Bassus albobasalis*	H003	Thailand: Phetchabun		KX431802	KX431721
*Bassus albobasalis*	H092	Thailand: Trang		------	KX431720
*Bassus albobasalis*	H328	Thailand: Phetchabun		JQ763436	KX431722
*Bassus albocyclus*	H308	Thailand: Phetchabun	paratype	------	KX431724
*Bassus albocyclus*	H349	Thailand: Chiang Mai	paratype	------	KX431725
*Bassus albocyclus*	H636	Thailand: Suphan Buri	holotype	ATRMK230-11	KX431723
*Bassus calculator*	H8008	Sweden: Stockholms län		------	KX431712
*Bassus mediatratus*	H015	Thailand: Chiang Mai	holotype	KX431816	KX431760
*Bassus nopachoni*	H577	Thailand: Kamphaeng	holotype	ATRMK223-11	KX431713
*Bassus pallidus*	H055	Thailand: Chanthaburi	holotype	------	KX431710
*Bassus* sp.	H376	Thailand: Phetchaburi		ATRMK204-11	KX431711
*Braunsia smithii*	H906	Thailand: Chiang Mai		ATRMK261-11	HQ667949
*Camptothlipsis lingualongis*	H1887	South Africa: Western Cape	paratype	ATRMK334-11	JN564494
*Camptothlipsis nigra*	H433	Thailand: Prachuap Khiri Khan		ATRMK430-11	HQ667951
*Camptothlipsis sheilae*	H664	Thailand: Kanchanaburi	holotype	ATRMK235-11	HQ667954
*Camptothlipsis* sp.	H162	Uganda: Homa Bay		------	KX431699
*Camptothlipsis* sp.	H2299	Congo: Pool		------	KX431698
*Chimaeragathis chrysoma*	H710	Thailand: Phetchaburi	holotype	ATRMK240-11	KX431738
*Chimaeragathis eurysoma*	H045	Thailand: Trang	paratype	KX431805	KX431736
*Chimaeragathis eurysoma*	H069	Thailand: Trang	paratype	KX431806	KX431737
*Chimaeragathis eurysoma*	H925	Thailand: Phetchaburi	holotype	ATRMK265-11	KX431735
*Chimaeragathis lohmani*	H072	Thailand: Trang	holotype	KX431807	KX431739
*Chimaeragathis lohmani*	H077	Thailand: Trang	paratype	KX431808	KX431740
*Cymagathis krikoma*	H290	Thailand: Chaiyaphum	paratype	ATRMK192-11	KX431701
*Gyragathis leucosoma*	H275	Thailand: Nakhon Ratchasima	holotype	KX431794	KX431700
*Leuroagathis paulbakeri*	H369	Thailand: Prachuap Khiri Khan	holotype	------	KX431709
*Liragathis baonai*	H360	Thailand: Nakhon Si Thammarat	holotype	ATRMK200-11	KX431705
*Liragathis damnai*	H397	Thailand: Chiang Mai	paratype	ATRMK210-11	KX431704
*Liragathis javana*	H283	Thailand: Trang		KX431795	KX431702
*Liragathis javana*	H628	Thailand: Phetchabun		ATRMK228-11	KX431703
*Neothlipsis parysae*	H4428	USA: KY	paratype	ATRMK364-11	KX431696
*Neothlipsis* sp.	H195	Thailand: Surat Thani		KP943607	KP943660
*Neothlipsis* sp.	H198	USA: KY		KX431793	KX431697
*Neothlipsis* sp.	H7618	Mexico: Yucatan		ATRMK403-11	KP943709
*Scabagathis emilynadeauae*	H033	Thailand: Trang	holotype	KX431792	KX431695
*Trochantagathis marshi*	H067	Thailand: Trang		KX431809	KX431742
*Trochantagathis marshi*	H089	Thailand: Trang		KX431811	KX431745
*Trochantagathis marshi*	H1851	Thailand: Trang		------	KX431744
*Trochantagathis marshi*	H281	Thailand: Trang		KX431810	KX431743
*Trochantagathis marshi*	H765	Thailand: Ubon Ratchathani		ATRMK242-11	KX431741
*Trochantagathis marshi*	H799	Thailand: Suphan Buri		------	KX431746
*Trochantagathis marshi*	H965	Thailand: Nakhon Si Thammarat		ATRMK266-11	KX431747
*Xanthagathis mellisoma*	H060	Thailand: Trang		KX431812	KX431749
*Xanthagathis mellisoma*	H145	Thailand: Phetchabun		------	KX431748
*Xanthagathis mellisoma*	H286	Thailand: Chaiyaphum		ATRMK191-11	KX431751
*Xanthagathis mellisoma*	H348	Thailand: Chiang Mai		ATRMK199-11	KX431750
*Xanthagathis mellisoma*	H662	Thailand: Phetchaburi		ATRMK234-11	KX431752
*Zosteragathis contrastus*	H017	Thailand: Chiang Mai		KX431828	KX431783
*Zosteragathis contrastus*	H056	Thailand: Trang		KX431834	KX431790
*Zosteragathis contrastus*	H094	Thailand: Chiang Mai		KX431833	KX431789
*Zosteragathis contrastus*	H100	Thailand: Chaiyaphum		KX431832	KX431787
*Zosteragathis contrastus*	H101	Thailand: Loei		KX431827	KX431781
*Zosteragathis contrastus*	H104	Thailand: Loei		------	KX431782
*Zosteragathis contrastus*	H142	Thailand: Nakhon Ratchasima		------	KX431779
*Zosteragathis contrastus*	H143	Thailand: Phetchabun		KX431829	KX431784
*Zosteragathis contrastus*	H144	Thailand: Phetchabun		KX431830	KX431785
*Zosteragathis contrastus*	H146	Thailand: Phetchabun		KX431831	KX431786
*Zosteragathis contrastus*	H149	Thailand: Phetchabun		KX431826	KX431780
*Zosteragathis contrastus*	H1855	Thailand: Chaiyaphum		ATRMK501-11	------
*Zosteragathis contrastus*	H603	Thailand: Surat Thani		ATRMK226-11	KX431791
*Zosteragathis contrastus*	H677	Thailand: Suphan Buri		------	KX431788
*Zosteragathis contrastus*	H985	Thailand: Kanchanaburi		------	KX431778
*Zosteragathis samensis*	H2418	Thailand: Prachuap Khiri Khan	holotype	ATRMK475-11	KX431775
*Zosteragathis samensis*	H973	Thailand: Prachuap Khiri Khan	paratype	ATRMK269-11	KX431774
*Zosteragathis* sp.	H065	Thailand: Trang		KX431803	KX431733
*Zosteragathis* sp.	H083	Thailand: Trang		KX431804	KX431734
*Zosteragathis* sp.	H091	Thailand: Sakon Nakhon		KX443589	KX431726
*Zosteragathis* sp.	H1859	Thailand: Phitsanulok		ATRMK329-11	KX431729
*Zosteragathis* sp.	H1860	Thailand: Surat Thani		ATRMK330-11	KX431731
*Zosteragathis* sp.	H239	Thailand: Trang		------	KX431732
*Zosteragathis* sp.	H492	Thailand: Phetchaburi		ATRMK217-11	KX431728
*Zosteragathis* sp.	H660	Thailand: Mae Hong Son		ATRMK233-11	KX431727
*Zosteragathis* sp.	H687	Thailand: Nakhon Si Thammarat		------	KX431730
*Zosteragathis* sp.	H016	Thailand: Chaiyaphum		KX431825	KX431776
*Zosteragathis* sp.	H080	Thailand: Chiang Mai		KX431814	KX431757
*Zosteragathis* sp.	H121	Thailand: Nong Bua Lam Phu		KX431822	KX431771
*Zosteragathis* sp.	H122	Thailand: Nong Bua Lam Phu		KX431823	KX431772
*Zosteragathis* sp.	H1625	Thailand: Chaiyaphum		ATRMK323-11	KX431754
*Zosteragathis* sp.	H1636	Thailand: Ubon Ratchathani		ATRMK325-11	KX431770
*Zosteragathis* sp.	H1858	Thailand: Chiang Mai		ATRMK328-11	KX431777
*Zosteragathis* sp.	H236	Thailand: Chiang Mai		KX431813	KX431756
*Zosteragathis* sp.	H237	Thailand: Lampang		KX431815	KX431758
*Zosteragathis* sp.	H279	Thailand: Ubon Ratchathani		KX431824	KX431773
*Zosteragathis* sp.	H473	Thailand: Phetchaburi		ATRMK216-11	KX431708
*Zosteragathis* sp.	H598	Thailand: Mae Hong Son		ATRMK225-11	KX431768
*Zosteragathis* sp.	H650	Thailand: Phetchabun		ATRMK232-11	KX431769
*Zosteragathis* sp.	H689	Thailand: Suphan Buri		ATRMK238-11	KX431755
*Zosteragathis* sp.	H989	Thailand: Phetchaburi		ATRMK271-11	KX431759
